# Tuning SpyTag–SpyCatcher mutant pairs toward orthogonal reactivity encryption†
†Electronic supplementary information (ESI) available: Supporting figures, molecular cloning, protein expression and purification protocols, protein sequences and other molecular characterizations. See DOI: 10.1039/c7sc02686b



**DOI:** 10.1039/c7sc02686b

**Published:** 2017-07-19

**Authors:** Yajie Liu, Dong Liu, Wei Yang, Xia-Ling Wu, Luhua Lai, Wen-Bin Zhang

**Affiliations:** a Key Laboratory of Polymer Chemistry & Physics of Ministry of Education , Center for Soft Matter Science and Engineering , College of Chemistry and Molecular Engineering , Peking University , Beijing 100871 , P. R. China . Email: wenbin@pku.edu.cn; b School of Life Sciences , Tsinghua University , Beijing 100084 , P. R. China; c BNLMS , Peking-Tsinghua Center for Life Sciences , Academy for Advanced Interdisciplinary Studies (AAIS) , Center for Quantitative Biology , State Key Laboratory for Structural Chemistry of Unstable and Stable Species , College of Chemistry and Molecular Engineering , Peking University , Beijing 100871 , P. R. China . Email: lhlai@pku.edu.cn

## Abstract

Genetically encoded covalent peptide tagging technology, such as the SpyTag–SpyCatcher reaction, has emerged as a unique way to do chemistry with proteins. Herein, we report the reactivity engineering of SpyTag–SpyCatcher mutant pairs and show that distinct reactivity can be encrypted for the same reaction based on protein sequences of high similarity. Valuable features, including high selectivity, inverse temperature dependence and (nearly) orthogonal reactivity, could be achieved based on as few as three mutations. This demonstrates the robustness of the SpyTag–SpyCatcher reaction and the plasticity of its sequence specificity, pointing to a family of engineered protein chemistry tools.

## Abstract 2

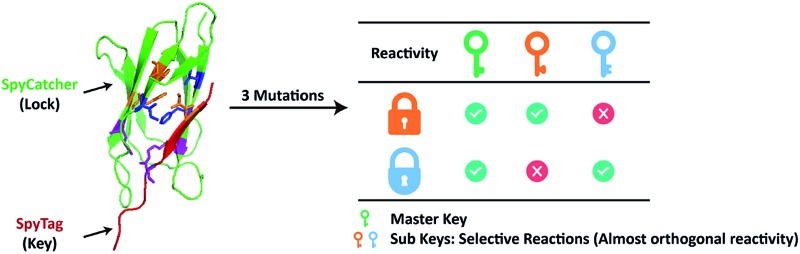
Distinct chemical reactivity, in addition to other valuable features, can be encrypted within protein sequences that differ by only three mutations.

## Introduction

**Fig. 1:**
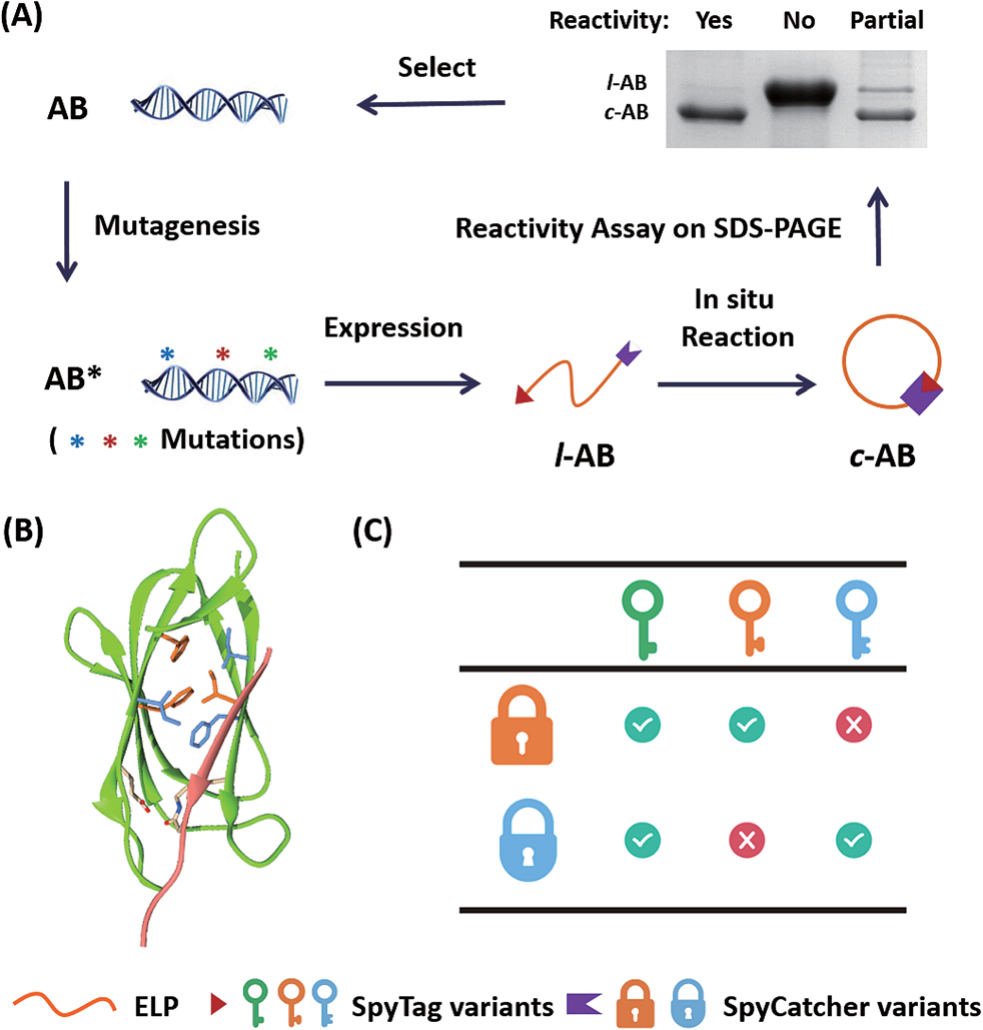
(A) Directed evolution of the SpyTag–SpyCatcher reactive pairs by sequential site-saturation mutagenesis and reactivity assay by SDS-PAGE analysis. (B) Crystal structures of the SpyTag and SpyCatcher complex (PDB: 4MLS). SpyTag is shown in pink and SpyCatcher is shown in green. The catalytic isopeptide triad is shown as pale sticks, the three key mutation sites are colored orange, and the other key residues in the hydrophobic pocket are colored blue. (C) By considering SpyTag as the key and SpyCatcher as the lock, we aimed to find a master key that can open all locks (*i.e.* forming covalent isopeptide bonds) and a pair of orthogonal sub-keys/sub-locks.

In recent years, covalent peptide tagging technology has attracted numerous research interests as a “molecular superglue” possessing “infinite” affinity to protein partners.^1–5^ Based on isopeptide-forming pilin subunits,^6^ a few reactive pairs have been developed, including isopeptag-N and pilin-N,^7^ SpyTag and SpyCatcher,^8^ SnoopTag and SnoopCatcher^9^ and SdyTag and SdyCatcher.^10^ Such reactions are considered genetically encodable, allowing one to program the post-translational modification of proteins in cells and expand the protein backbone topology beyond the linear configuration.^11–13^ Not only have cyclic, tadpole-like and other branched proteins been prepared,^11^ but direct cellular syntheses of protein catenanes and star proteins have also been demonstrated.^12,13^ Covalent tagging is thus an “iron grip” for synthetic biology,^5^ offering tremendous opportunities in applications such as preparing all-protein-based hydrogels,^14^ making “living” materials,^15^ engineering synthetic vaccines,^16^ controlling protein cellular locations^17^ and membrane protein activity^18^ and enhancing sortase efficiency.^19^ The extraordinary reactivity of SpyTag–SpyCatcher chemistry is reminiscent of “click” chemistry in materials science.^20^ Nevertheless, unlike functional groups in synthetic molecules, whose reactivity is defined mostly by their intrinsic properties, the reactivity of such peptide–protein pairs is instead defined by sequences and the resulting folded structures. Such a prerequisite precludes its application in nonaqueous environments or other denaturing conditions, but it also promises a family of peptide–protein reactive pairs with diverse features, distinct specificity and stimuli-responsive reactivity based on the same structural scaffold.^21^ In other words, even for the same reaction, the reactivity might be encoded orthogonally. In this contribution, we report the rational design and directed evolution of SpyTag–SpyCatcher reactive pairs toward orthogonal reactivity with sequences of high similarity (Fig. 1). We show that valuable features, including high selectivity, reversed temperature dependence and (nearly) orthogonal reactivity, could be achieved on the SpyTag–SpyCatcher scaffold with as few as three mutations.

## Results and discussion

### Evolving the reactive pairs

Directed evolution is a powerful technique in protein engineering to improve protein stability and to adapt proteins to new substrates, as well as to create new protein functions.^22–24^ The key lies in the generation of genetic diversity and the identification of protein variants with the desired properties.^25^ To date, no methods have been developed to direct the evolution of chemically reactive peptide–protein pairs. This is non-trivial, as this is a two component system and it is not easy to distinguish chemical bonding from strong physical association under the normal conditions used for screening. It was reported that the SpyTag–SpyCatcher reaction allowed the *in vivo* cyclization of an elastin-like protein (ELP) (AB).^11^ We envisioned that the ratio between cyclized (c-AB) and linear (l-AB) proteins, as shown in SDS-PAGE, would represent their reactivity under the conditions of expression (*i.e.* 16 °C in *Escherichia coli*) (Fig. 1A). Although this is not a high throughput method, it still provides a convenient way to screen the variants’ reactivity.

To begin with, we targeted the key residues within 5 Å around SpyTag in the complex (PDB: 4MLS) as the protein–protein interaction interface, which is critical for tuning the specificity and efficiency of the reactive pairs (Fig. 1B). To generate genetic diversity, we performed sequential focused site-saturation mutagenesis on each of them.^26^ In the first round, we introduced mutations onto SpyTag at the position of isoleucine (Ile3) to abolish its reactivity. The I3W mutant was found to yield almost exclusively the linear product, indicating poor reactivity (Fig. S1†). Based on this, we constructed a library of variants with site-saturation mutagenesis on SpyCatcher, and screened for restored reactivity in the subsequent rounds of directed evolution (see Fig. S1 and Table S1† for typical screening results). Finally, we found that a F77V, F94A mutant of SpyCatcher (B_VA_) partially restores the reactivity with A_W_ (Table S1†). Using this strategy, we aimed to achieve orthogonal reactivity encryption within the same protein scaffold with as few mutations as possible. If we make an analogy between the SpyTag–SpyCatcher reaction and the “lock-and-key” concept, we hope to find a master key that opens all locks (*i.e.* forming isopeptide bonds), and a pair of orthogonal sub-keys/sub-locks for selective functionalization (Fig. 1C).

### *In vitro* reactivity assay

**Fig. 2:**
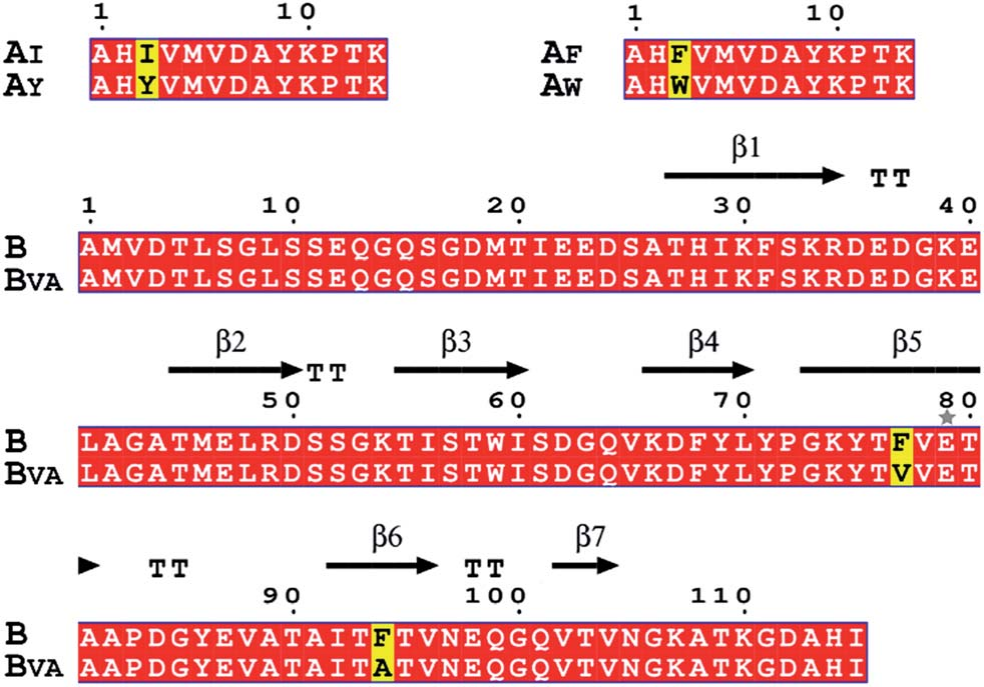
Amino acid sequence alignments of SpyTag, SpyCatcher and their mutants. Key mutations are colored yellow. The secondary structure of the SpyTag–SpyCatcher complex (PDB: 4MLS) is shown for comparison. The picture was generated by ESPript 3.0.

To gain a full picture of the reactivity profile between the SpyTag mutants and the final SpyCatcher mutant (B_VA_), the SpyTag mutants were individually fused with a green fluorescent protein (A_X_-GFP), where X stands for the mutated amino acid (see Fig. S2 for the sequences and S3 for the mass spectra†). SpyCatcher (B) and the final mutant, B_VA_, were also cloned and expressed separately (see Fig. 2 and S4 for the sequences and S5 for the mass spectra†) for *in vitro* reactivity assay with A_X_-GFP.

**Fig. 3:**
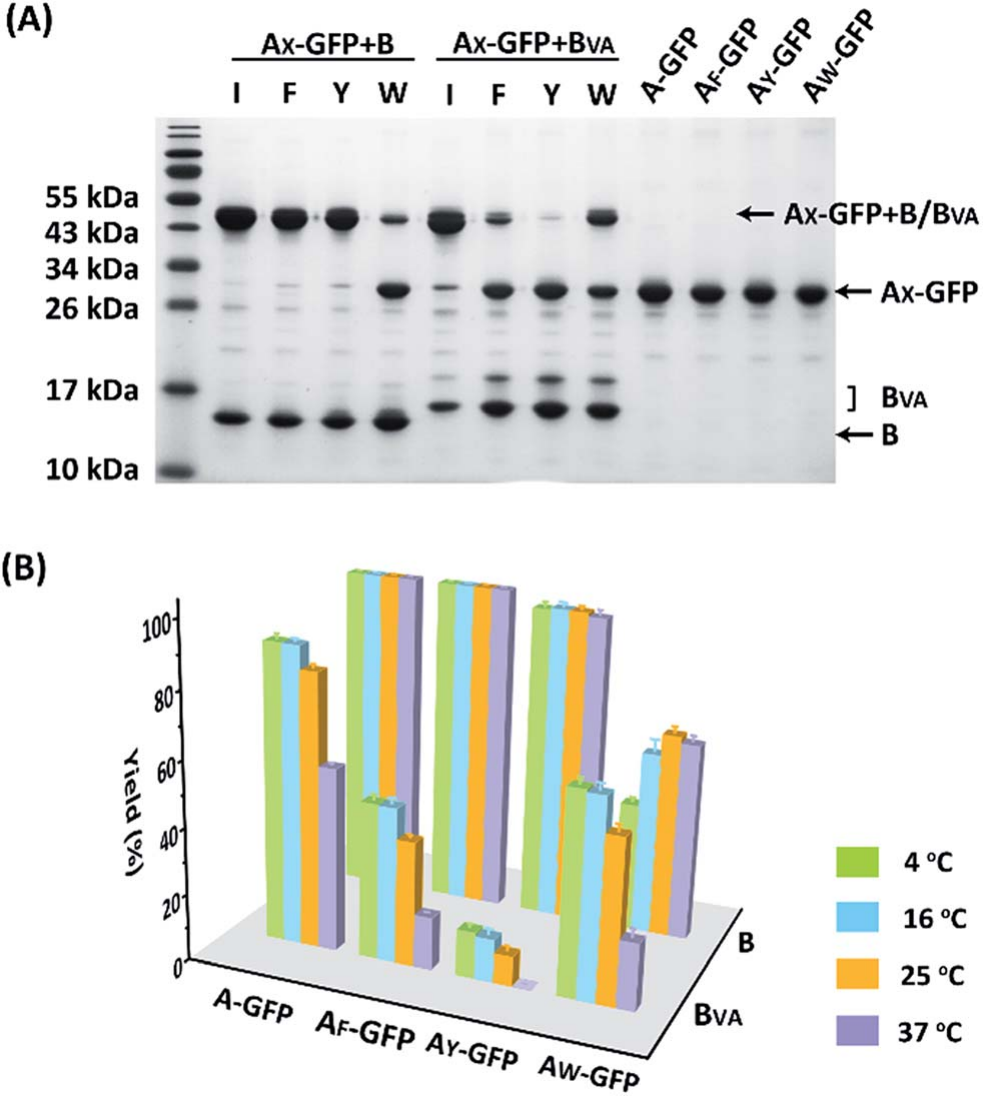
(A) SDS-PAGE analysis of the reaction products of A_X_-GFP and B or B_VA_ at 4 °C for 5 hours; (B) yields after reaction at 4, 16, 25 and 37 °C for 5 hours.

The reaction was carried out in PBS at pH 7.4, at different temperatures, at a concentration of 40 μM for A_X_-GFP and 80 μM for B or B_VA_. The kinetics were followed by taking aliquots at designated times and the yields were determined by SDS-PAGE and gel densitometry (Fig. 3 and S6–S12†). In general, the SpyTag–SpyCatcher reaction remains the best of its kind. Mutations on either side seem to compromise the reactivity. The kinetics toward B are slowed down slightly from A-GFP to A_F_-GFP, more significantly from A-GFP to A_Y_-GFP, and dramatically from A-GFP to A_W_-GFP. For B_VA_, the trend is similar, but the kinetics are much slower than those for B. Surprisingly, its reactivity is almost nullified toward A_Y_-GFP, but is restored toward A_W_-GFP, even though the side chain of Trp is considerably larger. Specifically, we compared the results after reaction at 4 °C for 5 hours, as shown in Fig. 3A. SpyTag behaves like a master key with excellent reactivity toward both B and B_VA_. The mutant’s reactivity toward SpyCatcher drops upon increasing the size of the side chains. Eventually, the yield between A_W_-GFP and B is merely ∼39%. While B_VA_ retains full reactivity with A-GFP, the yield drops to 47% for A_F_-GFP and 14% for A_Y_-GFP, but rises again to 60% for A_W_-GFP. The trend of reactivity is consistent with the *in vivo* cyclization results, but the yields seem a little higher because we purposely used a higher concentration of reactants and an excess of B or B_VA_ to drive the reaction forward. The possibility to manipulate the reactivity by the experimental conditions is an advantage for *in vitro* experiments. Interestingly, the temperature dependence of the reactivity is completely opposite for B and B_VA_ (Fig. 3B and S6†). This is best demonstrated in their reactions with A_W_-GFP. For B, the yield increases and plateaus at higher temperatures, but for B_VA_, the yield is promoted at lower temperatures. This suggests that they may have different mechanisms for reconstitution and reaction. From the current data, we deduced that (i) B_VA_ exhibits selective reactivity with A, but not A_Y_, whereas A_Y_ reacts well with B; (ii) at lower temperatures, A_W_ would preferentially react with B_VA_, while A_Y_ would selectively react with B, which is close to orthogonal.

**Fig. 4:**
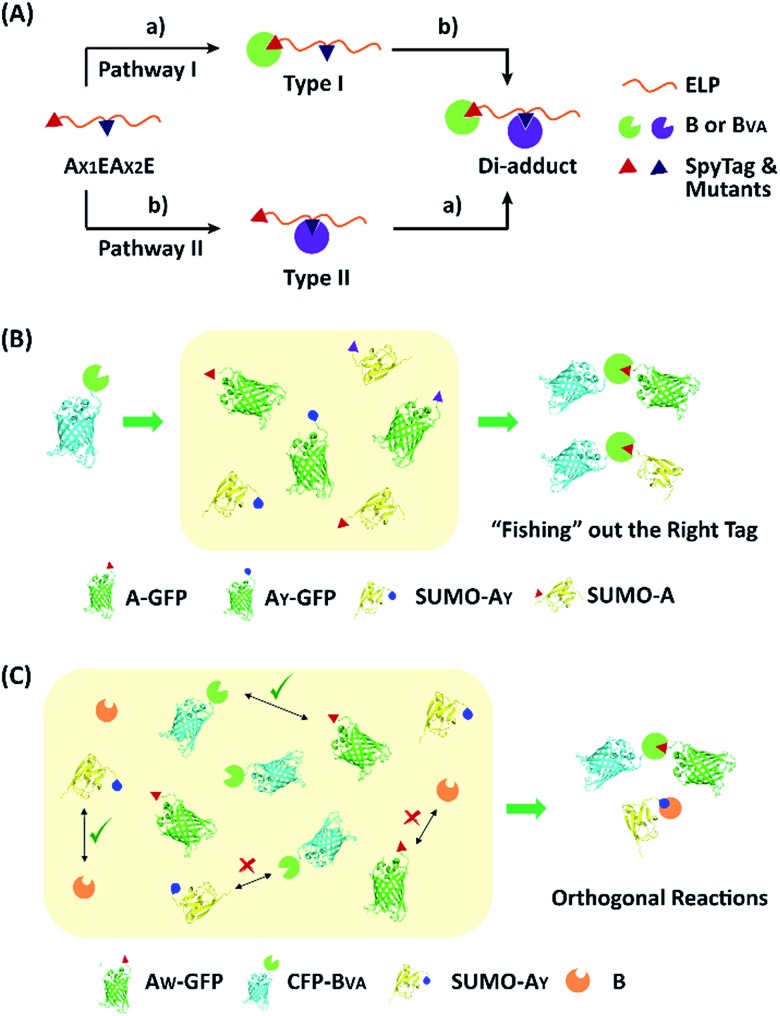
Experimental design to test the reaction selectivity and orthogonality: (A) the sequential reaction of A_X1_EA_X2_E with B or B_VA_ may proceed through two pathways; (B) B_VA_ could selectively react with A-GFP or SUMO-A but not A_Y_-GFP or SUMO-A_Y_; (C) orthogonal reactions lead to only two corresponding products from a complex mixture of reactive proteins.

To examine the selectivity of these mutant pairs, we designed a series of experiments. In the first one, elastin-like proteins (E) bearing A_Y_ (or A_W_) at the N-terminus and A (or A_Y_) in the middle of the chain were designed (A_X1_EA_X2_E), as well as their nonreactive controls, where the reactive Asp in either one of the SpyTag variants was changed to Ala (A_X1_′EA_X2_E or A_X1_EA_X2_′E) (Fig. S13–S16†). There are two possible pathways for their reaction with SpyCatcher and its mutant. Depending on the first reaction site, this would lead to type I and II intermediates (Fig. 4A). If selectivity permits, sequential functionalization could be performed on one protein scaffold to give distinct di-adducts. By design, the nonreactive mutants will trap the reaction at different intermediate states as controls. The second design was to use a B_VA_-functionalized cyan fluorescent protein (CFP-B_VA_) to selectively fish out the right tag from a pool of telechelic proteins containing different SpyTag mutants (such as A_X_-GFP and SUMO-A_X_) (the “tag-fishing” experiment is shown in Fig. 4B and S17–S20†). Finally, to rigorously test the orthogonality, we mixed multiple reactive proteins in one pot and determined the product distribution (Fig. 4C).

### Achieving selective reactions

**Fig. 5:**
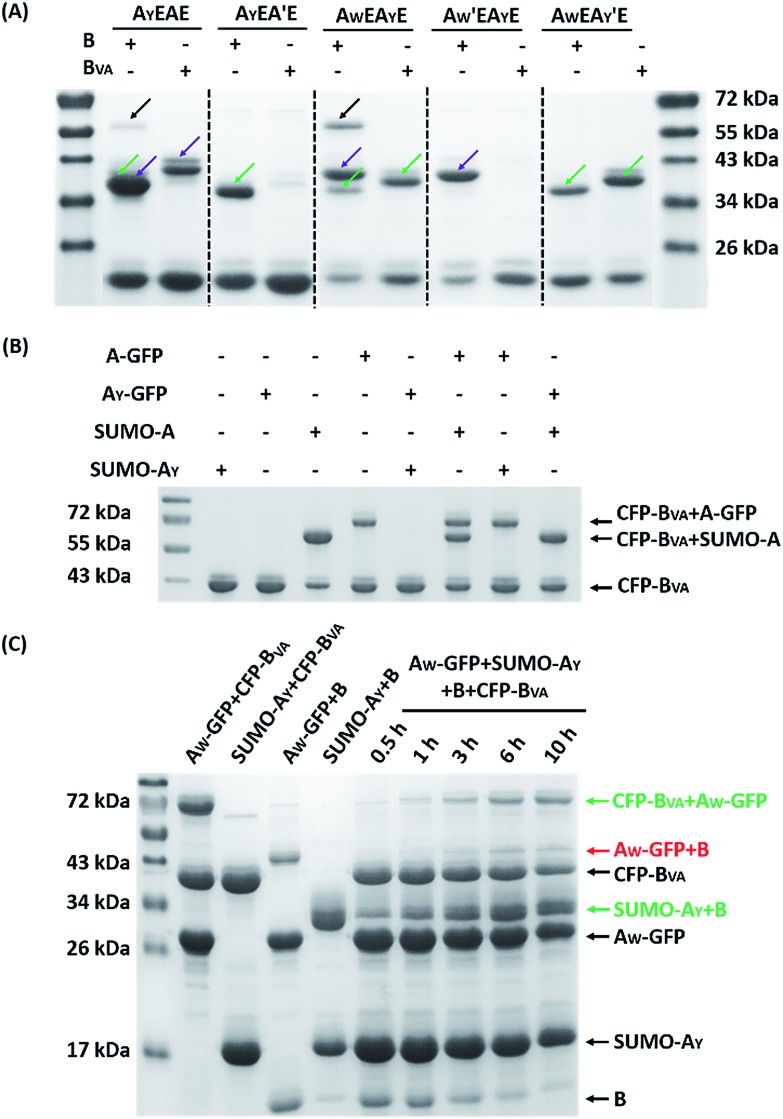
(A) SDS-PAGE analysis of the reaction products of A_X1_EA_X2_E and B or B_VA_ at 4 °C for 12 hours ([A_X1_EA_X2_E] : [B or B_VA_] = 15 μM : 15 μM); the green arrows point to the type I mono-adduct, the purple arrows point to the type II mono-adduct, and the black arrows point to the di-adduct. (B) SDS-PAGE analysis of the reaction products between CFP-B_VA_ and a mixture of proteins containing SpyTag or its mutant ([CFP-B_VA_] : [A_X_-GFP or SUMO-A_X_] = 30 μM : 30 μM). No reaction occurred between CFP-B_VA_ and any protein containing A_Y_. (C) SDS-PAGE analysis of the products from the one-pot reactions in a mixture of A_W_-GFP, SUMO-A_Y_, CFP-B_VA_ and B at different times at 4 °C ([CFP-B_VA_ or B] : [A_W_-GFP or SUMO-A_Y_] = 30 μM : 60 μM). The cross-reaction product formed at later stages in very small amounts.

**Fig. 6:**
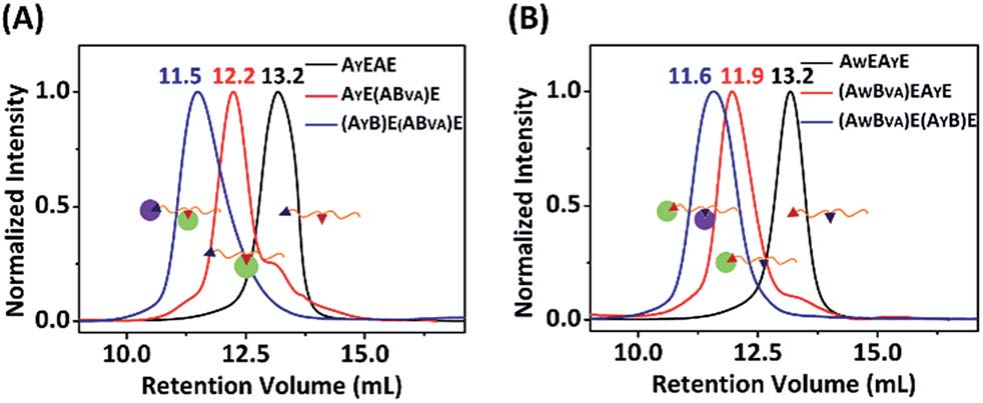
SEC overlay of the products at each stage from the sequential reaction of A_Y_EAE (left) or A_W_EA_Y_E (right), firstly with B_VA_ and then with B. The numbers above the peaks denote the retention volume of each peak.

Fig. 5A shows the reaction products between A_X1_EA_X2_E and B or B_VA_ at 4 °C at 15 μM each for 12 hours. By comparison with the control reactions, it is evident that B reacts efficiently with both A and A_Y_ while B_VA_ only reacts with A, but rarely with A_Y_ (Fig. S21†). The reaction using a B_VA_-functionalized ELP (EB_VA_) gives similar results (Fig. S21†). Although previous model experiments show slight residual reactivity between B_VA_ and A_Y_ (Fig. 3), it seems that this is inhibited tremendously in the presence of A due to the distinct kinetic difference between A and A_Y_, even though A_Y_ is placed at a more accessible terminal site. This feature should be useful for controlling the functionalization sites and preparing proteins with complex topology. As an example, we sequentially reacted A_Y_EAE firstly with B_VA_ and then with B. The size exclusion chromatography (SEC) overlay of the products at two stages clearly shows the sequential progression of the reactions (Fig. 6), and the identity of the products was corroborated by MALDI-TOF mass spectra and SDS-PAGE analysis (Fig. S22 and S23†). This excellent selectivity was also reflected in the “tag-fishing” experiments. We used CFP-B_VA_ to react with A_X_-GFP and/or SUMO-A_X_ at 4 °C in PBS buffer (pH 7.4) in a 1 : 1 molar ratio at a concentration of 30 μM each for 5 hours. The SDS-PAGE analysis clearly shows that no products formed from a mixture of CFP-B_VA_ with any A_Y_-containing protein, whereas a mixture of CFP-B_VA_ with any SpyTag-containing protein yielded the corresponding products (Fig. 5B and S24A†). The ability to distinguish the reactivity of peptide tags that differ by only one amino acid is remarkable.

### Tuning toward orthogonal reactions

The ability to encode information about chemical reactions into protein sequences greatly adds to the diversity of reactions and reactivity control. Even for the same type of reaction, there may be multiple, mutually orthogonal ways for reactivity encryption. While reactive pairs developed from completely different domains are intrinsically orthogonal to each other, orthogonality may also arise from the same ancestor domain.

Since A_W_ and A_Y_ possess opposite reactivity to B and B_VA_, we proceeded to examine how orthogonal they are to each other. We firstly reacted A_W_EA_Y_E, as well as the A_W_′EA_Y_E and A_W_EA_Y_′E controls, with B or B_VA_ at 4 °C for 12 hours, at a molar ratio of 1 : 1 with a concentration of 15 μM each, and analyzed the results by SDS-PAGE. It was clear that both A_W_ and A_Y_ are reactive toward B, and there are two bands for the mono-adducts and one for the di-adduct (Fig. 5A). By comparison of the products with the controls, we deduced that the lower band of the mono-adducts is the type I mono-adduct and the higher band is the type II mono-adduct. There is a much higher selectivity for A_Y_ in the middle over A_W_ at the chain end. Considering that the terminal location of A_W_ is kinetically much more advantageous, the intrinsic selectivity for A_Y_ should actually be even higher. To prove this, we constructed A_Y_EA_W_E and reacted it with B or B_VA_ under identical conditions. The results show that the reaction of A_Y_EA_W_E with B gives almost exclusively the type I product and very little di-adducts, indicating a very high selectivity (Fig. S21C†). On the other hand, for B_VA_, the selectivity for A_W_ over A_Y_ is excellent. No products form from the reaction between A_Y_ and B_VA_ at all, while the yield from the reaction between B_VA_ and A_W_ is decent for A_W_EA_Y_E when A_W_ is at a terminal site, and poor for A_Y_EA_W_E when A_W_ is in the middle (Fig. S21C†). The good selectivity encouraged us to also pursue sequential functionalization on A_W_EA_Y_E, firstly with B_VA_ and then with B. The products at each stage were characterized by MALDI-TOF mass spectra and SEC (Fig. 6, S22 and S23†). The results are similar to the previous case, except that the intermediate is a type I mono-adduct in this case instead of a type II mono-adduct. The two intermediates have different retention volumes, with type I being smaller (11.9 *vs.* 12.2 mL). This is consistent with the expanded linear-chain-like topology of the type I mono-adduct and the compact star-like topology of the type II mono-adduct, confirming again the highly selective functionalization.

A strict evaluation of the orthogonality would be from multiple reactants in one pot. It was hoped that the reactions would proceed in parallel in one pot without mutual interference. We thus mixed A_W_-GFP, SUMO-A_Y_, CFP-B_VA_ and B at a concentration of 30 μM for the SpyCatcher variants and 60 μM for the SpyTag variants at 4 °C. Control reactions were run by mixing only two reactive proteins under identical conditions (Fig. 5C, lanes 2–5). A time course for the one-pot reaction was recorded (Fig. 5C, lanes 6–10). This clearly shows that the major products are the desired products, CFP-B_VA_:A_W_-GFP and B:SUMO-A_Y_. There is only very little cross-reacted product of B:A_W_-GFP appearing at the late stages (∼6 hours). No cross-reacted product of CFP-B_VA_:SUMO-A_Y_ was observed (Fig. S24†). Therefore, A_W_/B_VA_ and A_Y_/B could be viewed as two mutant pairs with nearly orthogonal reactivity at shorter times and lower temperatures. Even considering the product distribution after 10 hours of reaction, B still has ∼9.1-fold selectivity for SUMO-A_Y_ over A_W_-GFP, and A_W_-GFP still exhibits ∼5.5-fold selectivity for CFP-B_VA_ over B. Considering that the two reactive pairs only differ by three amino acids, this result is impressive. It is also worth noting that the reactivity may vary depending on the location of the reactive domains and the 3D structure of the fusion proteins, which may or may not promote the selectivity of the reactions.

To understand the reaction selectivity, we built structural models of the mutant pairs using RosettaDesign (Fig. S25†).^27,28^ When the I3W mutation is introduced into SpyTag, the bulky side chain of Trp clashes with the original residues on B, which leads to a calculated binding free energy as high as 534.99 REU (Rosetta energy units, an arbitrary energy unit based on the Rosetta score function).^29^ By changing the two Phe residues on B to smaller residues (Val and Ala), B_VA_ could better accommodate the Trp residue of A_W_, as demonstrated by the much lower calculated binding free energy of –36.14 REU for A_W_:B_VA_. On the other hand, the I3Y mutation in SpyTag retains its reactivity with SpyCatcher by forming good hydrophobic interactions between A_Y_ and B with a packing score of 0.758,^30^ while the interactions between A_Y_ and B_VA_ are significantly deteriorated, as reflected by the much lower packing score of 0.683. Although the detailed molecular mechanism affecting the reactivity remains to be illustrated by techniques like crystallography, the trend predicted by computational studies is generally consistent with the experimental results. Aided by computational studies and rational design, we are continuing to improve the orthogonality and reactivity of the mutant pairs along this line.

## Conclusions

In summary, we have developed mutants of SpyTag–SpyCatcher reactive pairs with engineered reactivity and selectivity through directed evolution. With only two mutations, B_VA_ exhibits distinct reactivity profiles compared to B. The inverse temperature dependence of the reactivity suggests that they may adopt different reconstitution mechanisms. SpyTag is like a master key, reactive with both B and B_VA_. A_Y_ is like a sub-key that only “opens” the lock B rather than B_VA_. In contrast, A_W_ possesses dramatically reduced reactivity with B but still exhibits decent reactivity with B_VA_ at lower temperatures. The reactivity between A_Y_/B and A_W_/B_VA_ is thus close to orthogonal with some minor cross-reaction between A_W_ and B. Although the promiscuity is not completely removed, the ability to engineer reactivity with minimum sequence variation (as few as three mutations) opens up new avenues in the ever-expanding “iron grip” toolbox for synthetic biology. It also promises a family of genetically encoded and engineered peptide–protein chemistry tools, much like what the fluorescent protein family has offered to the community.
