# Supplementary material for: Tuning SpyTag–SpyCatcher mutant pairs toward orthogonal reactivity encryption
Source: Chem Sci. 2017 Jul 19;8(9):6577–82. doi: 10.1039/c7sc02686b (PMC5627348; doi:10.1039/c7sc02686b)
Supplement: Supplementary file 1 [file SC-008-C7SC02686B-s001.pdf]

Electronic Supplementary Material (ESI) for Chemical Science.
This journal is © The Royal Society of Chemistry 2017

Electronic Supporting Information

for

Tuning SpyTag-SpyCatcher Mutant Pairs toward Orthogonal Reactivity Encryption

Yajie Liu,[1, §] *Dong Liu,*[1, §] *Wei Yang,*[2, §] *Xia-Ling Wu,*[1] *Luhua Lai,*[*, 3] *Wen-Bin Zhang*[*,1]

[1] Key Laboratory of Polymer Chemistry & Physics of Ministry of Education, Center for Soft Matter Science and Engineering, College of Chemistry and Molecular Engineering, Peking University, Beijing 100871, P. R. China

[2] School of Life Sciences, Tsinghua University, Beijing 100084, P. R. China

[3] BNLMS, Peking−Tsinghua Center for Life Sciences, Academy for Advanced Interdisciplinary Studies (AAIS), Center for Quantitative Biology, State Key Laboratory for Structural Chemistry of Unstable and Stable Species, College of Chemistry and Molecular Engineering, Peking University, Beijing 100871, P. R. China

[§] These authors contributed equally to the work.

E-mail: wenbin@pku.edu.cn; lhlai@pku.edu.cn

Experimental Section

DNA Construction. All oligonucleotide primers were ordered from Invitrogen. Sequence encoding SpyTag, SpyCatcher and ELP are used as reported before.[1] The GFP used in this paper is a variant devoid of methionine in sequence and the plasmid containing its gene was kindly given by Prof. David A. Tirrell at California Institute of Technology.[2] The plasmid containing SUMO was generously provided by Prof. Mark Howarth at the University of Oxford.[3] The plasmid containing CFP was ordered from Addgene.[4] All the genes were cloned into the bacterial expression vector pQE-80L (Qiagen Inc.) by standard restriction digestion and ligation protocols. Plasmids for each mutant were prepared following QuickChange® Site Directed Mutagenesis Protocol.[5] All DNA sequences were confirmed by direct sequencing.

Protein Expression and Purification. The recombinant plasmids were used to transform *Escherichia coli* strain BL21 for expression. The starter culture was prepared by inoculating 10 mL of 2XYT broth (containing 100 μg/mL ampicillin) with a single colony carrying recombinant plasmid and allowed to grow overnight at 37 ℃ and 220 rpm. The overnight culture was used to inoculate 1 L of 2XYT broth containing 100 μg/mL ampicillin and grown at 37 °C until $OD_{600\ nm}$ reached 0.7–1.0. Isopropyl-β-D-thiogalactopyranoside (IPTG) was added to a final concentration of 1 mM to induce target protein expression at 16 ℃. After a further 20 h growth at 220 rpm shaking, cells were pelleted by spinning at 5000g for 20 min at 4 ℃. The cell pellets were lysed by ultrasonication under native conditions (50 mM NaH_2PO_4, 300 mM NaCl, 10 mM imidazole, pH = 8.0). Then, the supernatant was collected by centrifugation (30 min × 25000 g at 4 °C). The protein was purified as described in the Qiagen Expressionist™ using 50% Ni-NTA slurry. The supernatant lysate was mixed with a 50% Ni-NTA slurry and agitated by rotator at 4 °C for 1 h. The mixture was then loaded into an empty column,

washed by wash buffer (50 mM NaH_2PO_4, 300 mM NaCl, 20 mM imidazole, pH = 8.0) for several column volumes and then eluted by elution buffer (50 mM NaH_2PO_4, 300 mM NaCl, 250 mM imidazole, pH = 8.0). Elution was pooled and further purified by Superdex 200 increase 10/300 GL column in an ÄKTA FPLC system (GE Healthcare, Inc.) using PBS (137 mM NaCl, 2.7 mM KCl, 10 mM Na_2HPO_4, 2 mM KH_2PO_4, pH = 7.4) as the mobile phase at a flow rate of 0.5 mL/min. Target peak was pooled and stored at -20 ^{o}C for further characterization. The protein yields were approximately 20-25 mg/L.

Protein Characterization. Sodium dodecyl sulfate polyacrylamide gel electrophoresis (SDS-PAGE) was performed to determine the apparent molecular weight of each protein. Size exclusion chromatography was performed on a Superdex 200 increase 10/300 GL column with ÄKTA FPLC system (GE Healthcare, Inc.). PBS (pH = 7.4) was used as the mobile phase at a flow rate of 0.5 mL/min. Matrix-assisted laser desorption ionization time-of-flight mass spectrometry (MALDI-TOF MS) was conducted on a MALDI TOF/TOF 5800 (AB Sciex, USA) mass spectrometer with sinapic acid as the matrix. Protein quantification was performed on a nanodrop (IMPLEN P33) for samples purified by SEC. Gel densitometry analysis was performed with ImageQuant TL software on Typhoon FLA9500 (GE Healthcare, Inc.) for SDS-PAGE images.

Reactivity Assay. All reaction assay was carried out in PBS buffer (pH = 7.4). The reactivity profile was obtained by reacting A_X-GFP and B (or B_{VA}) at 4 ^{o}C for 5 hours. The molar ratio between A_X-GFP and B (or B_{VA}) is 1:2 and the concentration of A_X-GFP is 40 μM. The temperature dependence experiments were carried out under identical conditions at 4, 16, 25, and 37 ^{o}C, respectively. The time course experiments were performed under identical conditions and aliquots were taken at different time points and boiled with denaturing buffer to quench the reaction for SDS-PAGE analysis. The

selectivity experiments were performed by mixing $A_YEAE/A_YEA'E$ and $B/B_{VA}/EB_{VA}$ at 4 °C for 12 hours (the molar ratio is 1:1 at the concentration of 15 μM each) and by mixing CFP-B_{VA} with A_X-GFP and/or SUMO-A_X at 4 °C for 5 hours (the molar ratio is 1:1 at the concentration of 30 μM each). To evaluate the orthogonality of the reactions, the experiments were performed by mixing $A_WEA_YE/A_WEA_Y'E/A_W'EA_YE$ and $B/B_{VA}/EB_{VA}$ at 4 °C for 12 hours at a molar ratio of 1:1 and a concentration of 15 μM each. The one-pot orthogonal reactions were carried out and by mixing together A_W-GFP, SUMO-A_Y, CFP-B_{VA} and B at 4 °C with a concentration of 30 μM for CFP-B_{VA} and B and 60 μM for A_W-GFP and SUMO-A_Y and taking out samples at designated times for analysis by SDS-PAGE. For sequential functionalization experiments, the telechelic proteins (A_YEAE or A_WEA_YE) was first reacted with B_{VA} at 4 °C at the molar ratio of 1:10 for 12 hours at a concentration of A_YEAE of 10 μM. The product was separated by SEC and characterized by MALDI-TOF mass spectrum and SDS-PAGE. Then, the isolated product was reacted with B at 4 °C at the molar ratio of 1:5 for 5 hours. The final product was also separated by SEC for analysis.

Computational Analysis. The interaction between SpyTag and SpyCatcher is vital for placing the reaction residue at optimal position and orientation for restoring the catalytic activity and at the same time providing hydrophobic environment for the reaction.[6] It is thus critical to have proper interactions for reaction to occur. We used RosettaRemodel[7] to introduce mutations to structure of wild type SpyTag and SpyCatcher complex (PDB code: 4MLS)[8] to build the structure models of the reaction pairs (A_Y/B, A_w/B_{VA}) and cross-reaction pairs(A_Y/B_{VA} and A_w/B) and optimize the structures using RosettaRelax.[9] The binding free energy of the mutant pairs was calculated using Rosetta InterfaceAnalyzer.[10]

(A)

1 MKGSSHHHHHHVDAH**I**VMV**D**AYKPTKLDGHGVGVPGVGVPGVGVPGEGVPGVGVPGVGVP
61 GVGVPGVGVPGEGVPGVGVPGVGVPGVGVPGVGVPGEGVPGVGVPGVGELYAVTGRGDSP
121 ASSAPIATSVPGVGVPGVGVPGEGVPGVGVPGVGVPGVGVPGVGVPGEGVPGVGVPGVGV
181 PGVGVPGVGVPGEGVPGVGVPGVGVPGGLLDIPTT*ENLYFQG*AMVDTLSGLSSEQGQSGD
241 MTIEEDSATHIKFS**K**RDEDGKELAGATMELRDSSGKTISTWISDGQVKDFYLYPGKYT**F**V
301 ETAAPDGYEVATAIT**F**TVNEQGQVTVNGKATKGDAHIDGPQGIWGQLE*

SpyTag sequence; SpyCatcher sequence; **Mutation site;** **Reactive amino acid;** *TEV site*

(B)

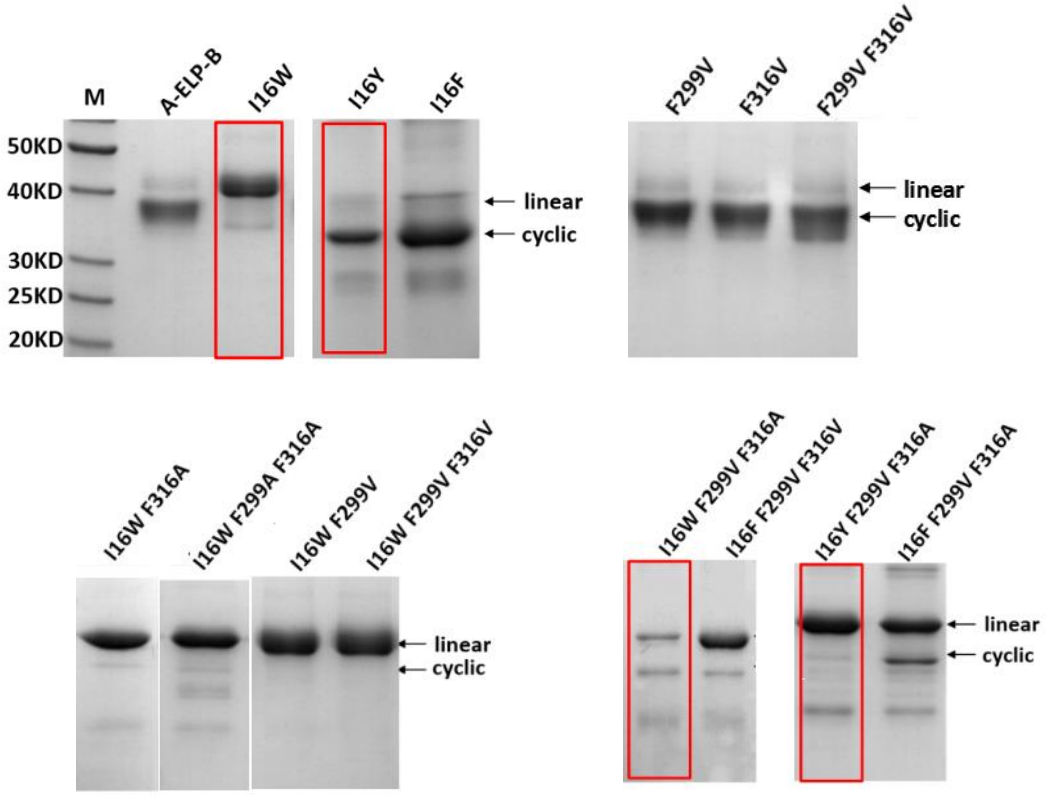

Figure S1. (A) The sequences of the telechelic protein AB and (B) the typical SDS-PAGE results of the expression products of AB mutants. The presence of cyclic AB (c-AB) suggests in vivo reaction whereas the linear AB suggests no reactivity. The numbers refer to the sequence in the AB construct.

Table S1. Summary of reactivity assay of the SpyTag-SpyCatcher mutants.

Mutants[a]	I16	F299	F316	Cyclic (Good Reactivity)	Linear (No Reactivity)
AB	I	F	F	+	
F299V		V		+	
F316V			V	+	
F299V F316V		V	V	+	
I16W	W				+
I16W F299V	W	V			+
I16W F316A	W		A		+
I16W F299V F316V	W	V	V		+
I16W F299V F316A	W	V	A	+	+
I16W F299A F316A	W	A	A		+
I16F	F			+	
I16F F299V F316V	F	V	V	+	+
I16F F299V F316A	F	V	A	+	+
I16Y	Y			+	
I16Y F299V F316A	Y	V	A		+

a): The numbers refer to the sequence in the AB construct.

A-GFP 275 a.a. MW=30683-18(H_2O)-2(2H)=30663

1 MKGSSHHHHHHVEASAHIVMVDAYKPTKVDSGGSGSMSKGEELFTGVVPILVELDGDVNG
61 HKFSVRGEGEGDATYGKITLKLICTTGKLPVPWPTLVTTCGYGVQCFARYPDHLKRHDFF
121 KSAFPEGYVQERTISFKDDGKFKTRAEVKFEGDTIVNRIKLKGIDFKEDGNILGHKLEYN
181 YNSHDVYITADKQKTGIKANFKIRHNVEDGSVQLADHYQQNTPIGDGPVRLPDNHYLLTQ
241 SVISKDPNEKRDHAVLHEFVTAAGITHGIDELYKK*

A_F-GFP 275 a.a. MW=30717-18(H_2O)-2(2H)=30697

1 MKGSSHHHHHHVEASAHFVMVDAYKPTKVDSGGSGSMSKGEELFTGVVPILVELDGDVNG
61 HKFSVRGEGEGDATYGKITLKLICTTGKLPVPWPTLVTTCGYGVQCFARYPDHLKRHDFF
121 KSAFPEGYVQERTISFKDDGKFKTRAEVKFEGDTIVNRIKLKGIDFKEDGNILGHKLEYN
181 YNSHDVYITADKQKTGIKANFKIRHNVEDGSVQLADHYQQNTPIGDGPVRLPDNHYLLTQ
241 SVISKDPNEKRDHAVLHEFVTAAGITHGIDELYKK*

A_Y-GFP 275 a.a. MW=30733-18(H_2O)-2(2H)=30713

1 MKGSSHHHHHHVEASAHYVMVDAYKPTKVDSGGSGSMSKGEELFTGVVPILVELDGDVNG
61 HKFSVRGEGEGDATYGKITLKLICTTGKLPVPWPTLVTTCGYGVQCFARYPDHLKRHDFF
121 KSAFPEGYVQERTISFKDDGKFKTRAEVKFEGDTIVNRIKLKGIDFKEDGNILGHKLEYN
181 YNSHDVYITADKQKTGIKANFKIRHNVEDGSVQLADHYQQNTPIGDGPVRLPDNHYLLTQ
241 SVISKDPNEKRDHAVLHEFVTAAGITHGIDELYKK*

A_W-GFP 275 a.a. MW=30756-18(H_2O)-2(2H)=30736

1 MKGSSHHHHHHVEASAHWVMVDAYKPTKVDSGGSGSMSKGEELFTGVVPILVELDGDVNG
61 HKFSVRGEGEGDATYGKITLKLICTTGKLPVPWPTLVTTCGYGVQCFARYPDHLKRHDFF
121 KSAFPEGYVQERTISFKDDGKFKTRAEVKFEGDTIVNRIKLKGIDFKEDGNILGHKLEYN
181 YNSHDVYITADKQKTGIKANFKIRHNVEDGSVQLADHYQQNTPIGDGPVRLPDNHYLLTQ
241 SVISKDPNEKRDHAVLHEFVTAAGITHGIDELYKK*

SpyTag sequence; GFP sequence; **Mutation site; Reactive amino acid**

Figure S2. Amino acid sequences of A_X-GFP. The calculation of GFP molecular weight takes into consideration the loss of one water molecule and two hydrogen atoms upon oxidation and maturation.

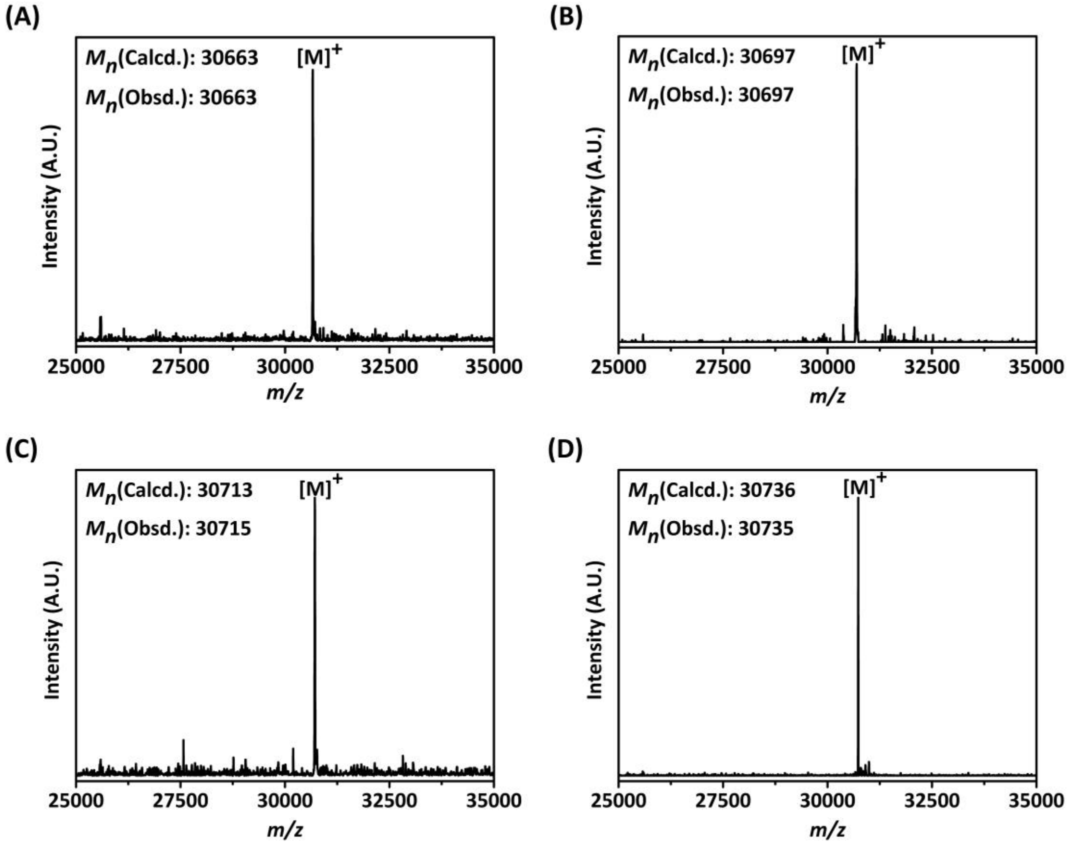

Figure S3. LC-MS spectra of A_X-GFP: (A) A-GFP; (B) A_F-GFP; (C) A_Y-GFP; (D) A_W-GFP.

B 134 a.a. MW=14645

1 MKGSSHHHHHHVDIPTT*ENLYFQG*AMVDTLSGLSSEQGQSGDMTIEEDSATHIKFS**K**RDE
61 DGKELAGATMELRDSSGKTISTWISDGQVKDFYLYPGKYT**F**VETAAPDGYEVATAIT**F**TV
121 NEQGQVTVNGKATK*

B$_{VA}$ 141 a.a. MW=15187

1 MKGSSHHHHHHVEASIPTT*ENLYFQG*AMVDTLSGLSSEQGQSGDMTIEEDSATHIKFS**K**R
61 DEDGKELAGATMELRDSSGKTISTWISDGQVKDFYLYPGKYT**V**VETAAPDGYEVATAIT**A**
121 TVNEQGQVTVNGKATKGDAHI*

SpyCatcher sequence; **Mutation site;** **Reactive amino acid;** *TEV site*

Figure S4. Amino acid sequences of SpyCatcher (B) and the mutant (B$_{VA}$).

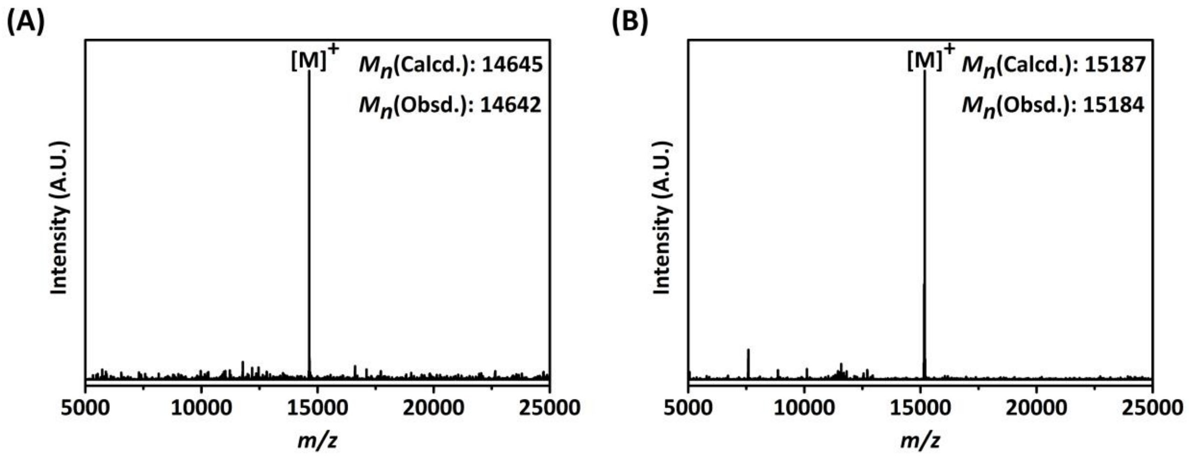

Figure S5. LC-MS spectra of SpyCatcher (B) and the mutant (B$_{VA}$).

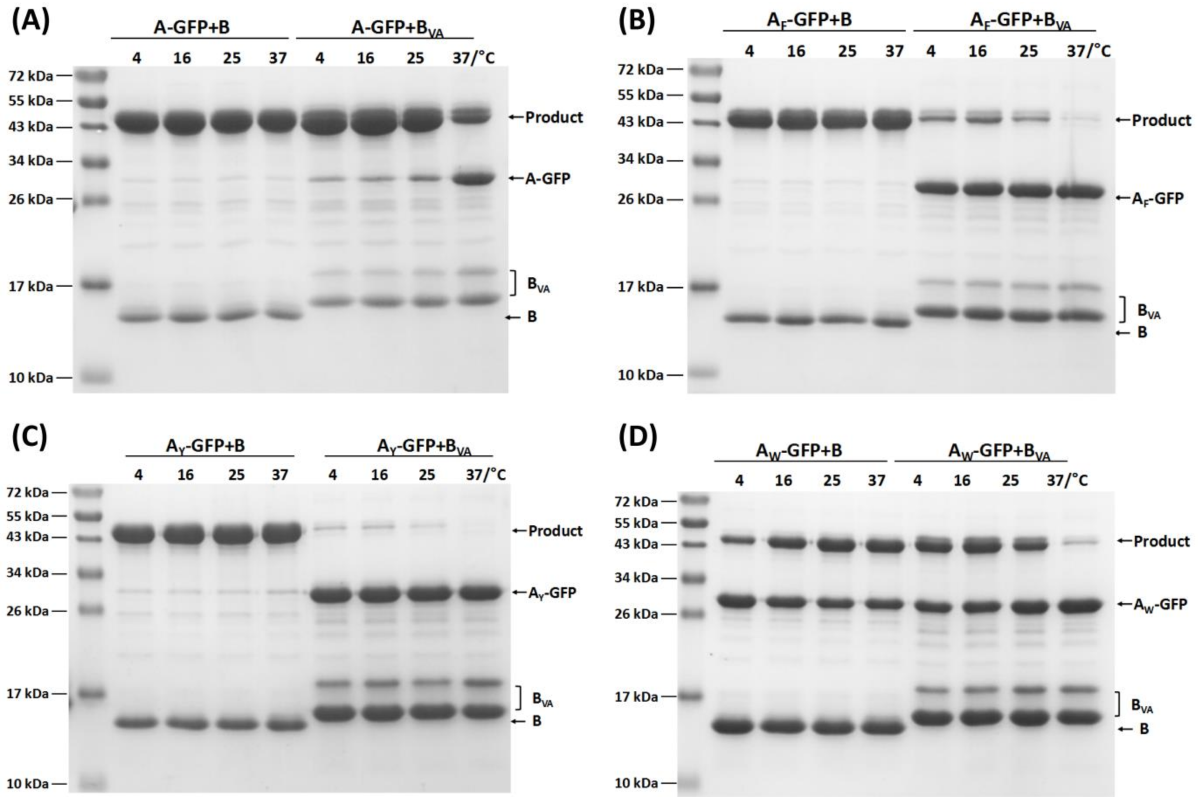

Figure S6. SDS-PAGE for screening the reactivity of B and B_{VA} with A_X-GFP at 4, 16, 25, and 37 ºC, respectively, for 5 hours at the molar ratio of 1:2. The concentration of A_X-GFP is 40 μM.

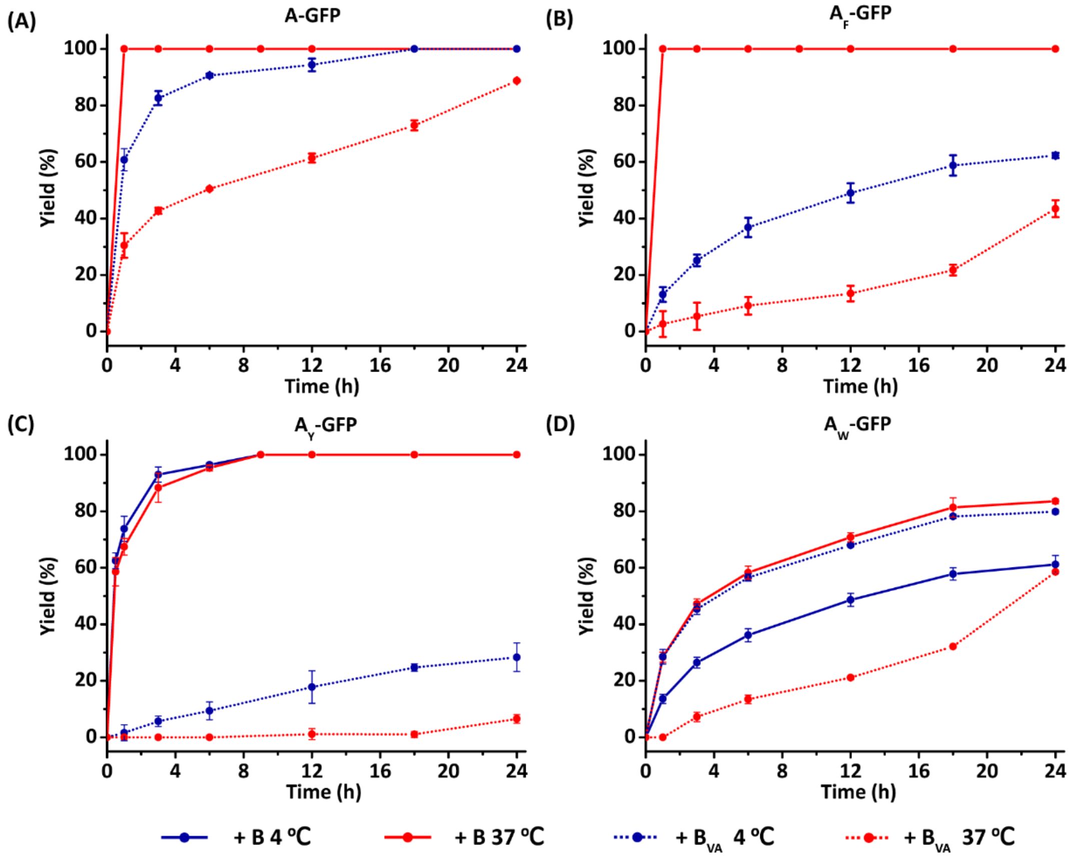

Figure S7. Time course of the reaction between A_X-GFP and B (or B_{VA}) at different temperatures. For all these reactions, the molar ratio between A_X-GFP and B (or B_{VA}) is 1:2 and the concentration of A_X-GFP is 40 μM.

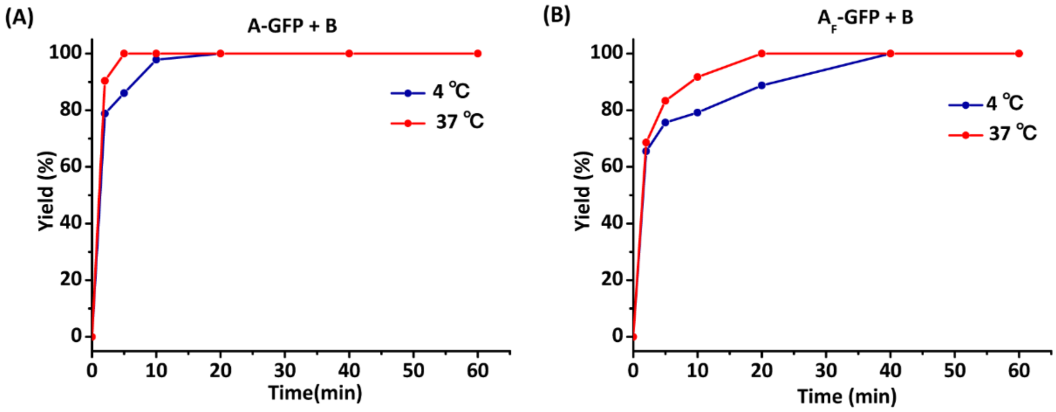

Figure S8. Time course graphs of A-GFP/A_F-GFP reacting with B at different temperatures within one hour. The molar ratio between A_X-GFP and B (or B_{VA}) is 1:2 and the concentration of A_X-GFP is 40 μM.

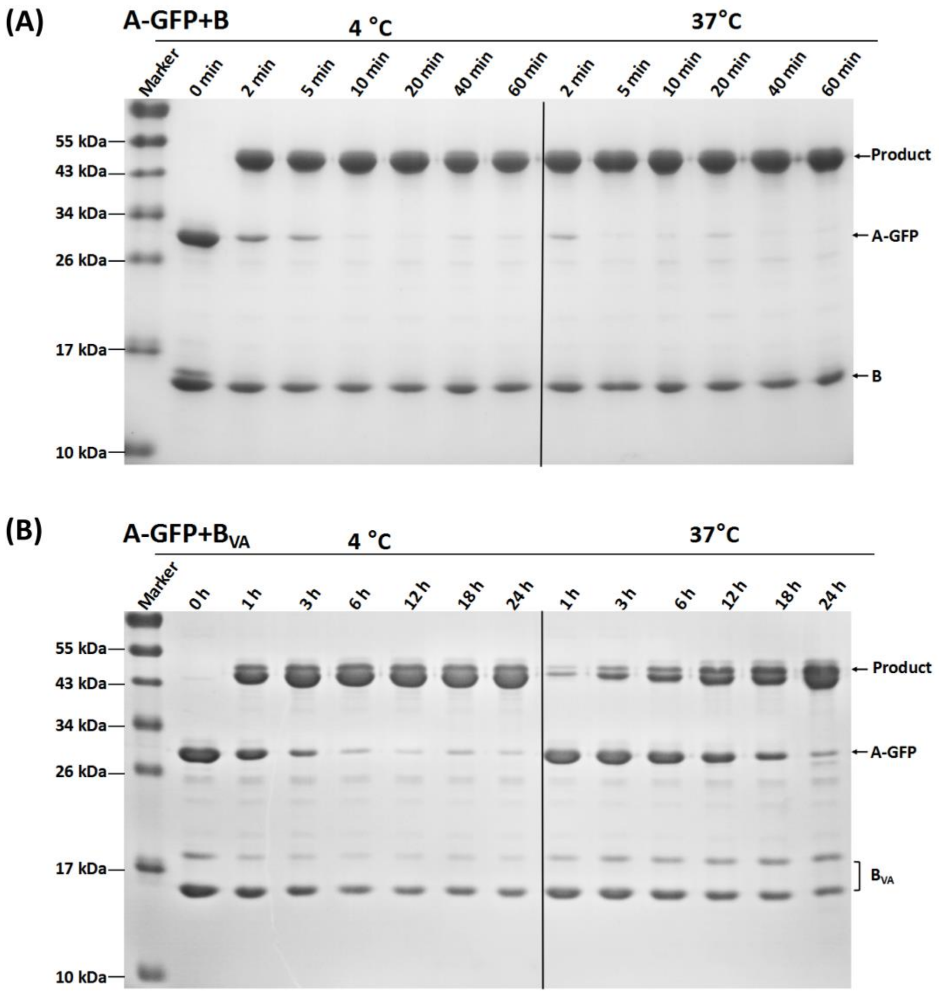

Figure S9. SDS-PAGE analysis of the time-course experiments for the reaction between A-GFP and SpyCatcher (B) or the mutant (B_{VA}) at 4 ºC and 37 ºC, respectively (the molar ratio is 1:2, and the concentration of A-GFP is 40 μM).

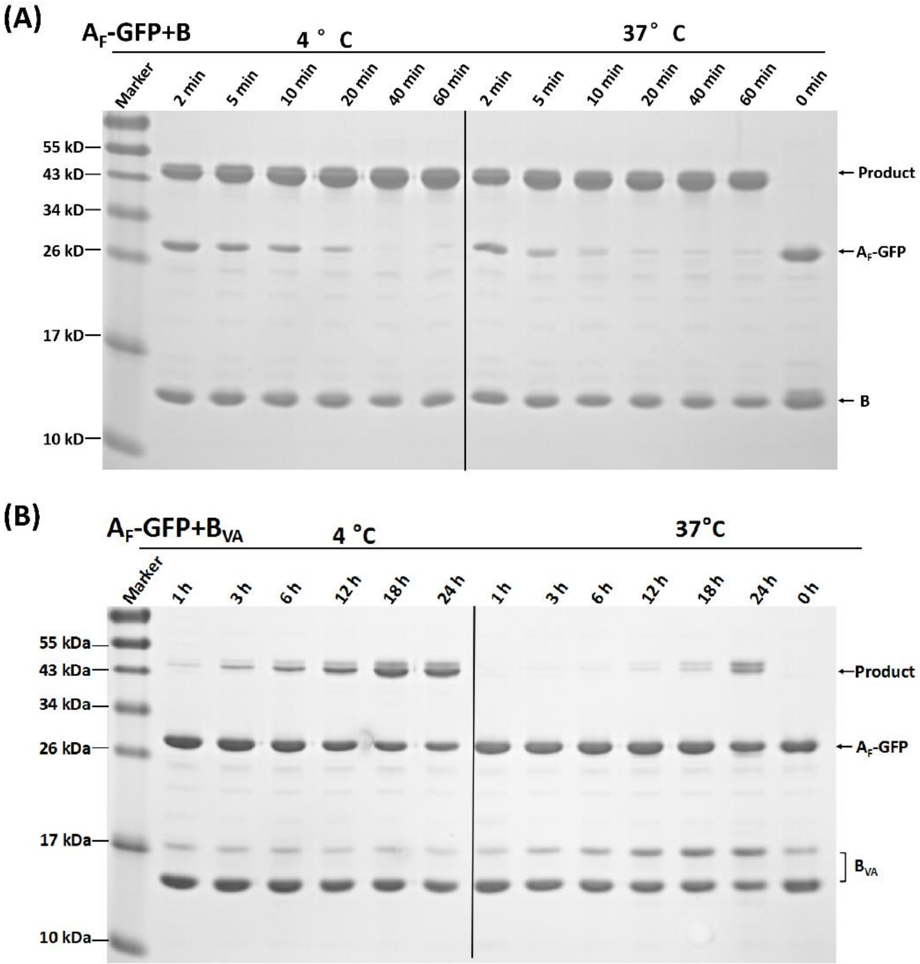

Figure S10. SDS-PAGE analysis of the time-course experiments for the reaction between A_F-GFP and SpyCatcher (B) or the mutant (B_{VA}) at 4 ºC and 37 ºC, respectively (the molar ratio is 1:2, and the concentration of A_F-GFP is 40 μM).

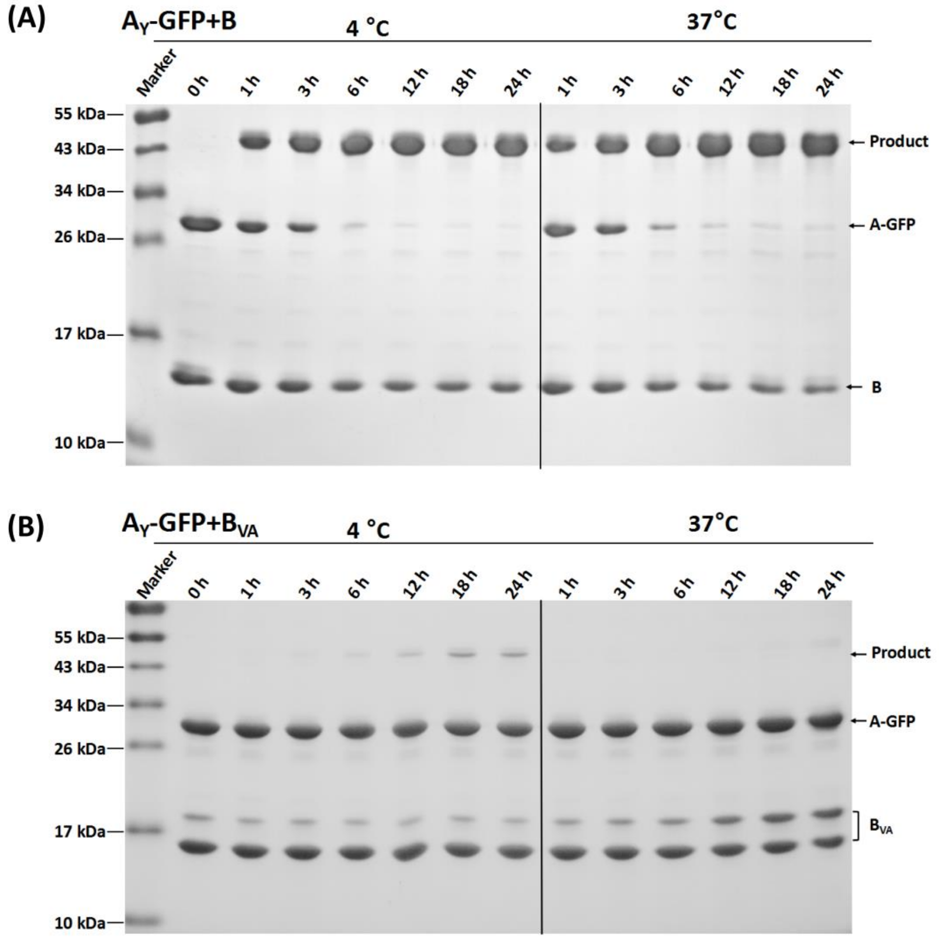

Figure S11. SDS-PAGE analysis of the time-course experiments for the reaction between A$_Y$-GFP and SpyCatcher (B) or the mutant (B$_{VA}$) at 4 ºC and 37 ºC, respectively (the molar ratio is 1:2, and the concentration of A$_Y$-GFP is 40 μM).

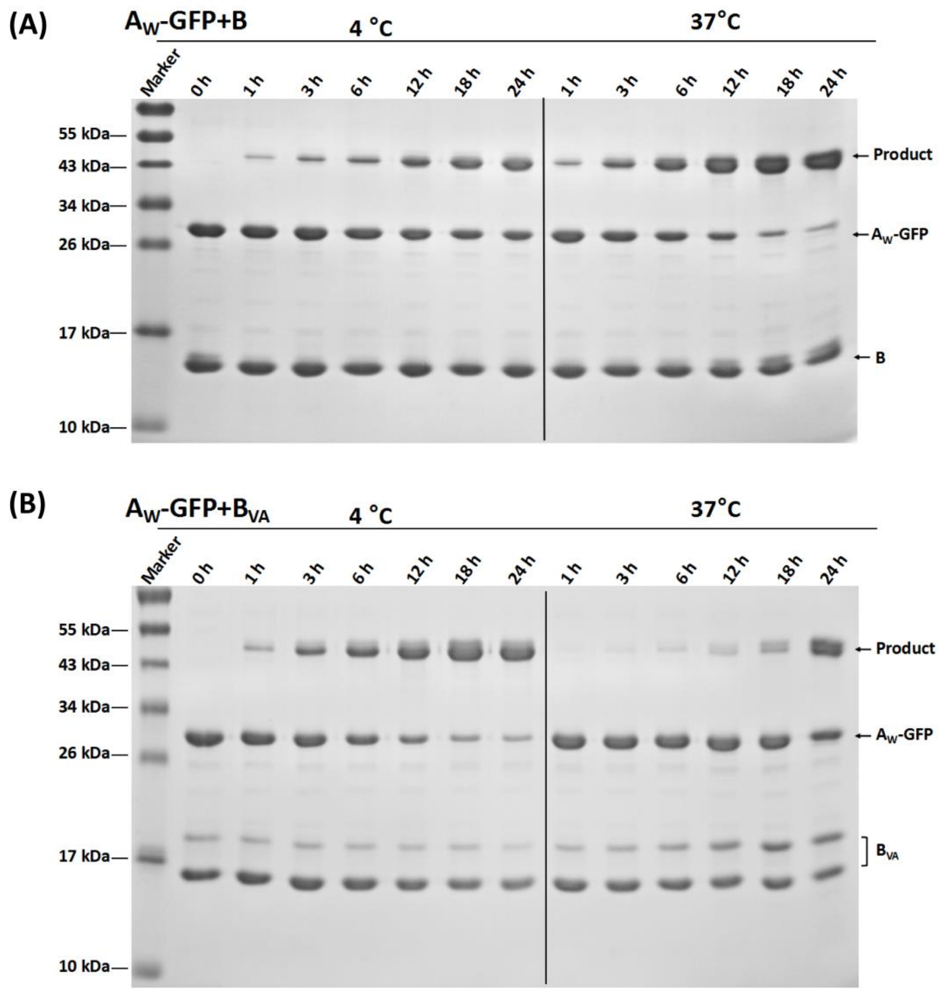

Figure S12. SDS-PAGE analysis of the time-course experiments for the reaction between A_W-GFP and SpyCatcher (B) or the mutant (B_{VA}) at 4 °C and 37 °C, respectively (the molar ratio is 1:2, and the concentration of A_W-GFP is 40 μM).

A$_Y$EAE 211 a.a. MW=19000

1 MKGSSHHHHHHVEASAHYVMVDAYKPTKVDGHGVGVPGVGVPGVGVPGEGVPGVGVPGVG
61 VPGVGVPGVGVPGEGVPGVGVPGVGVPGVGVPGVGVPGEGVPGVGVPGVGELAHIVMVDA
121 YKPTKTSVPGVGVPGVGVPGEGVPGVGVPGVGVPGVGVPGVGVPGEGVPGVGVPGVGVPG
181 VGVPGVGVPGEGVPGVGVPGVGVPGGLLDGP*

A$_Y$EA'E 211 a.a. MW=18956

1 MKGSSHHHHHHVEASAHYVMVDAYKPTKVDGHGVGVPGVGVPGVGVPGEGVPGVGVPGVG
61 VPGVGVPGVGVPGEGVPGVGVPGVGVPGVGVPGVGVPGEGVPGVGVPGVGELAHIVMVAA
121 YKPTKTSVPGVGVPGVGVPGEGVPGVGVPGVGVPGVGVPGVGVPGEGVPGVGVPGVGVPG
181 VGVPGVGVPGEGVPGVGVPGVGVPGGLLDGP*

SpyTag sequence; **Mutation site;** **Reactive amino acid;** **Mutation site with abolished reactivity**

Figure S13. Sequences of A$_Y$EAE and A$_Y$EA'E.

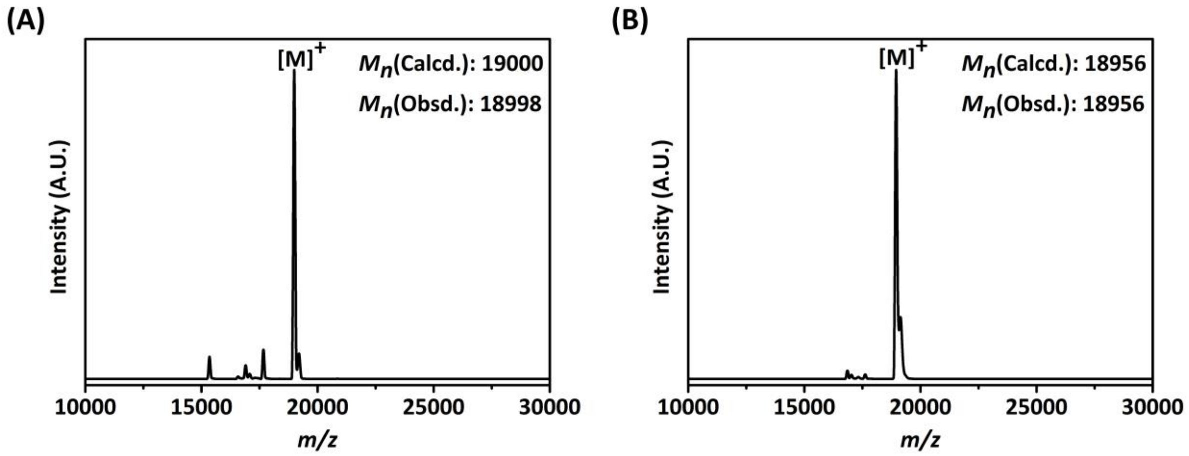

Figure S14. MALDI-TOF mass spectra of A$_Y$EAE and A$_Y$EA'E.

A$_W$EA$_Y$E 211 a.a. MW=19073

1 MKGSSHHHHHHVEASAHWVMVDAYKPTKVDGHGVGVPGVGVPGVGVPGEGVPGVGVPGVG
61 VPGVGVPGVGVPGEGVPGVGVPGVGVPGVGVPGVGVPGEGVPGVGVPGVGELAHYVMVDA
121 YKPTKTSVPGVGVPGVGVPGEGVPGVGVPGVGVPGVGVPGVGVPGEGVPGVGVPGVGVPG
181 VGVPGVGVPGEGVPGVGVPGVGVPGGLLDGP*

A$_W$EA$_Y$'E 211 a.a. MW=19029

1 MKGSSHHHHHHVEASAHWVMVDAYKPTKVDGHGVGVPGVGVPGVGVPGEGVPGVGVPGVG
61 VPGVGVPGVGVPGEGVPGVGVPGVGVPGVGVPGVGVPGEGVPGVGVPGVGELAHYVMVAA
121 YKPTKTSVPGVGVPGVGVPGEGVPGVGVPGVGVPGVGVPGVGVPGEGVPGVGVPGVGVPG
181 VGVPGVGVPGEGVPGVGVPGVGVPGGLLDGP*

A$_W$'EA$_Y$E 211 a.a. MW=19029

1 MKGSSHHHHHHVEASAHWVMVAAYKPTKVDGHGVGVPGVGVPGVGVPGEGVPGVGVPGVG
61 VPGVGVPGVGVPGEGVPGVGVPGVGVPGVGVPGVGVPGEGVPGVGVPGVGELAHYVMVDA
121 YKPTKTSVPGVGVPGVGVPGEGVPGVGVPGVGVPGVGVPGVGVPGEGVPGVGVPGVGVPG
181 VGVPGVGVPGEGVPGVGVPGVGVPGGLLDGP*

A$_Y$EA$_W$E 211 a.a. MW=19073

1 MKGSSHHHHHHVEASAHYVMVDAYKPTKVDGHGVGVPGVGVPGVGVPGEGVPGVGVPGVG
61 VPGVGVPGVGVPGEGVPGVGVPGVGVPGVGVPGVGVPGEGVPGVGVPGVGELAHWVMVDA
121 YKPTKTSVPGVGVPGVGVPGEGVPGVGVPGVGVPGVGVPGVGVPGEGVPGVGVPGVGVPG
181 VGVPGVGVPGEGVPGVGVPGVGVPGGLLDGP*

SpyTag sequence; **Mutation site; Reactive amino acid; Mutation site with abolished reactivity**

Figure S15. Sequences of A$_W$EA$_Y$E, A$_W$'EA$_Y$E, A$_W$EA$_Y$'E and A$_Y$EA$_W$E.

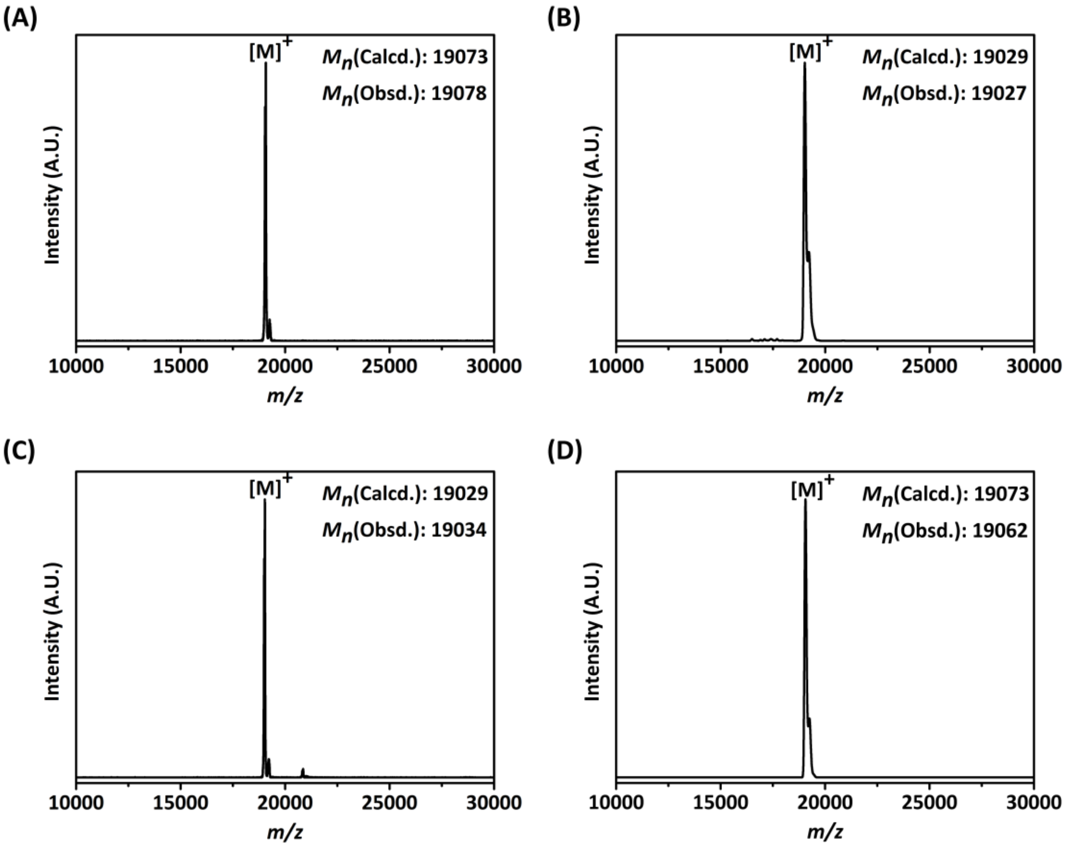

Figure S16. MALDI-TOF mass spectra of A_WEA_YE, A_W’EA_YE, A_WEA_Y’E and A_YEA_WE .

CFP-B$_{VA}$ 377 a.a. MW=41693-18(H_2O)-2(2H)=41673

1 MKGSSHHHHHHVEASMSKGEELFGGIVPILVELEGDVNGHKFSVSGEGEGDATYGKLTLK
61 FICTTGKLPVPWPTLVTTLTWGVQCFSRYPDHMKQHDFFKSVMPEGYVQERTIFFKDDGN
121 YKTRAEVKFEGDTLVNRIELKGIDFKEDGNILGHKLEYNYISHNVYITADKQKNGIKANF
181 KARHNITDGSVQLADHYQQNTPIGDGPVILPDNHYLSTQSALSKDPNEKRDHMVLLEFVT
241 AAGITHGMDELYKELAMVDTLSGLSSEQGQSGDMTIEEDSATHIKFS**K**RDEDGKELAGAT
301 MELRDSSGKTISTWISDGQVKDFYLYPGKYT**V**VETAAPDGYEVATAIT**A**TVNEQGQVTVN
361 GKATKGDAHIGTVEKKM*

ELP-B$_{VA}$ 336 a.a. MW=32195

1 MKGSSHHHHHHVDGHGVGVPGVGVPGVGVPGEGVPGVGVPGVGVPGVGVPGVGVPGEGVP
61 GVGVPGVGVPGVGVPGVGVPGEGVPGVGVPGVGELYAVTGRGDSPASSAPIATSVPGVGV
121 PGVGVPGEGVPGVGVPGVGVPGVGVPGVGVPGEGVPGVGVPGVGVPGVGVPGVGVPGEGV
181 PGVGVPGVGVPGGLLDIPTT*ENLYFQG*AMVDTLSGLSSEQGQSGDMTIEEDSATHIKFS**K**
241 RDEDGKELAGATMELRDSSGKTISTWISDGQVKDFYLYPGKYT**V**VETAAPDGYEVATAIT
301 **A**TVNEQGQVTVNGKATKGDAHIDGPQGIWGQLEWKK*

CFP sequence; SpyCatcher sequence; *TEV site;* **Mutation sites; Reactive amino acid**

Figure S17. Amino acid sequences of CFP-B$_{VA}$ and EB$_{VA}$. The calculation of CFP molecular weight takes into consideration the loss of one water molecule and two hydrogen atoms upon oxidation and maturation.

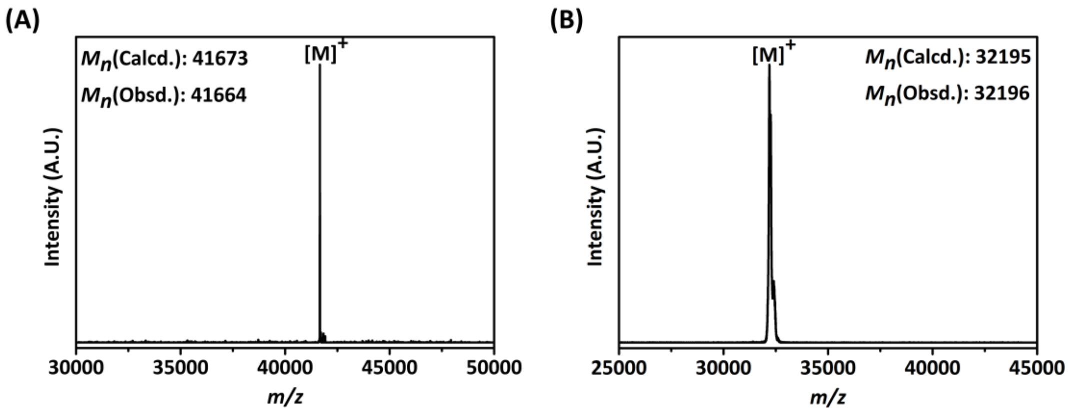

Figure S18. MALDI-TOF mass spectra of CFP-B$_{VA}$ and EB$_{VA}$.

SUMO-A 133 a.a. MW=15214

1 MKGSSHHHHHHVEASDSEVNQEAKPEVKPEVKPETHINLKVSDGSSEIFFKIKKTTPLRR
61 LMEAFAKRQGKEMDSLRFLYDGIRIQADQTPEDLDMEDNDIIEAHREQIGGLEAHIVMVD
121 AYKPTKGTVEKKM*

SUMO-A$_Y$ 133 a.a. MW=15264

1 MKGSSHHHHHHVEASDSEVNQEAKPEVKPEVKPETHINLKVSDGSSEIFFKIKKTTPLRR
61 LMEAFAKRQGKEMDSLRFLYDGIRIQADQTPEDLDMEDNDIIEAHREQIGGLEAHYVMVD
121 AYKPTKGTVEKKM*

SUMO sequence; SpyTag sequence; **Mutation site; Reactive amino acid**

Figure S19. Amino acid sequences of SUMO-A$_Y$ and SUMO-A.

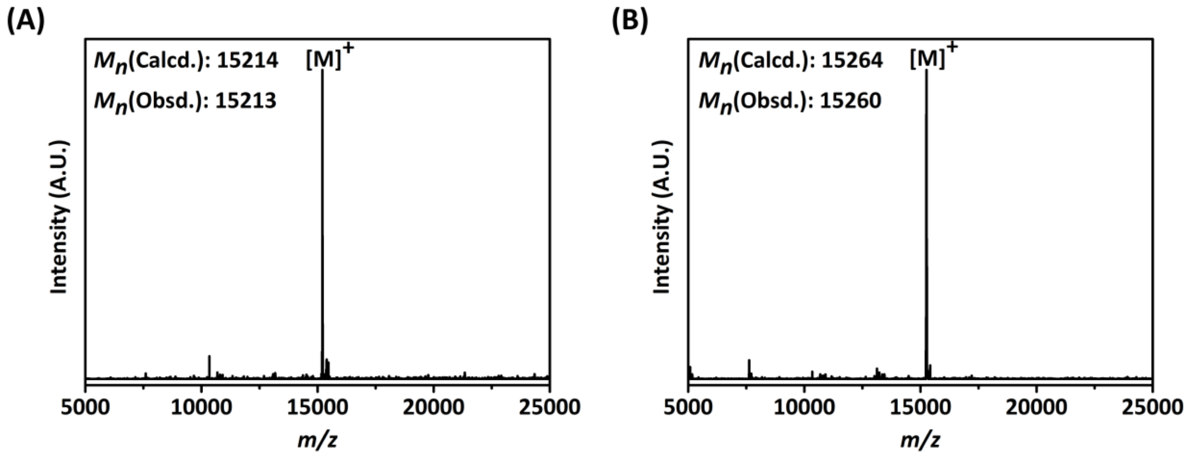

Figure S20. MALDI-TOF mass spectra of SUMO-A and SUMO-A$_Y$.

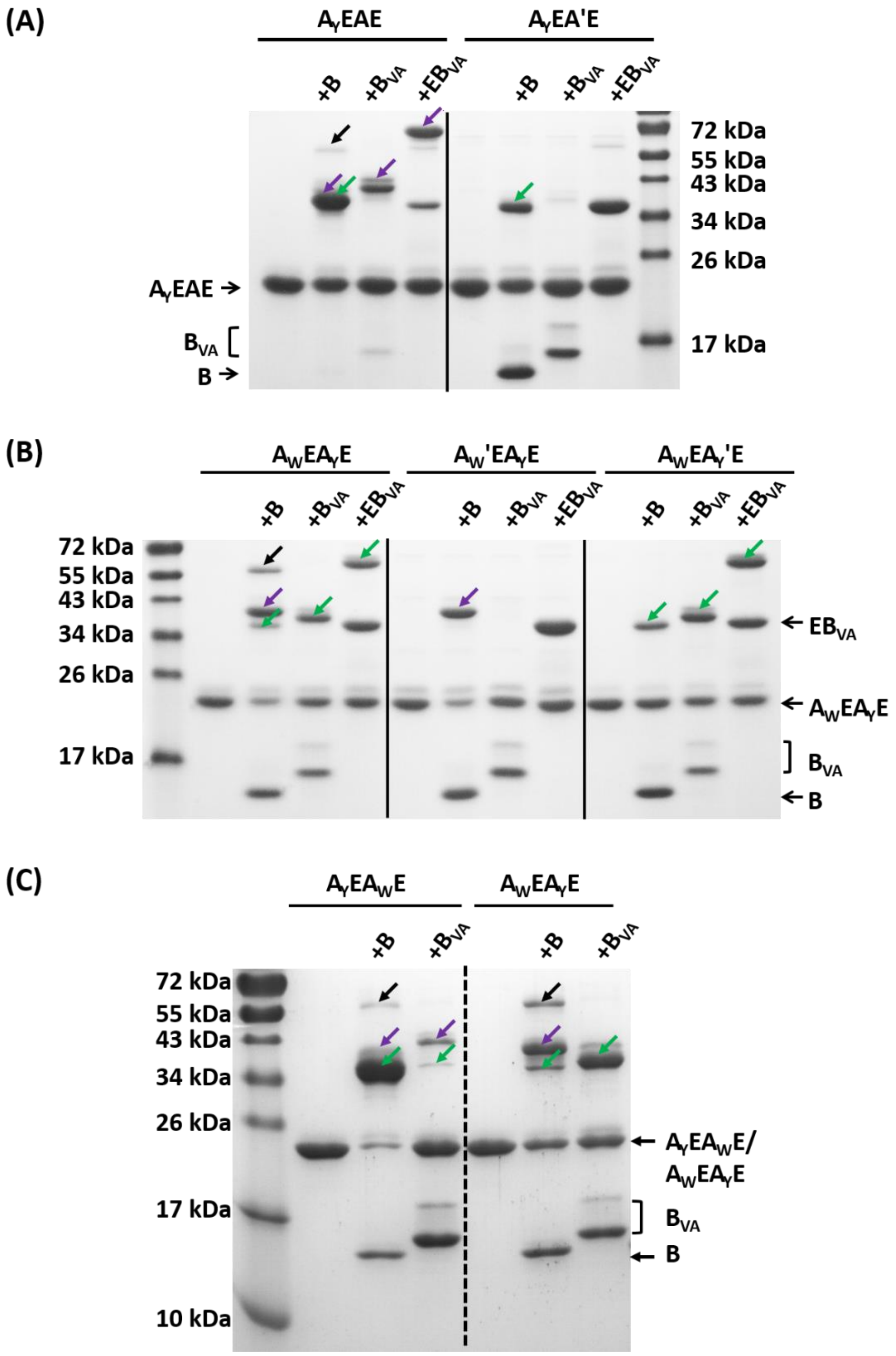

Figure S21. SDS-PAGE analysis of the reaction products: (A) between $A_YEAE/A_YEA'E$ and $B/B_{VA}/EB_{VA}$; (B) between $A_WEA_YE/A_WEA_Y'E/A_W'EA_YE$ and $B/B_{VA}/EB_{VA}$; and (C) between A_YEA_WE/A_WEA_YE and B/B_{VA}. The reaction was run at 4 °C for 12 hours with the molar ratio of 1:1 at the concentration of 15 μM each.

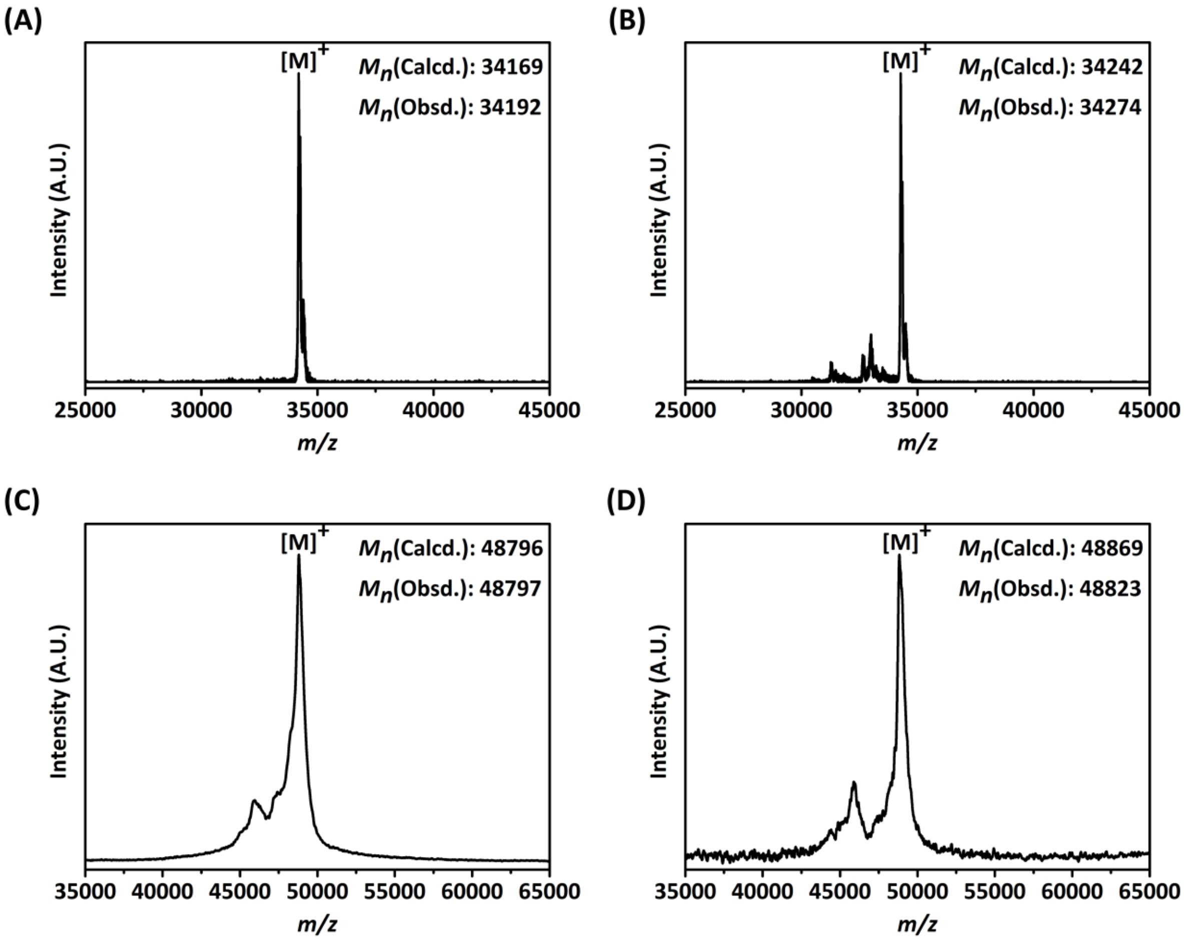

Figure S22. MALDI-TOF mass spectra of the reaction products of $A_YE(AB_{VA})E$, $(A_WB_{VA})EA_YE$, $(A_YB)E(AB_{VA})E$, and $(A_WB_{VA})E(A_YB)E$.

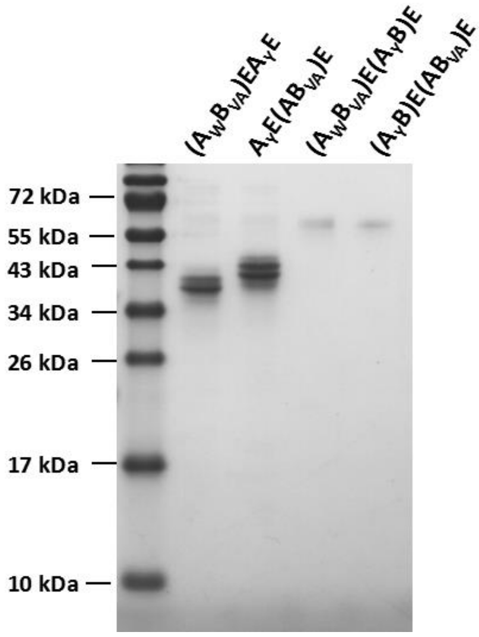

Figure S23. SDS-PAGE analysis of the reaction products of $(A_WB_{VA})EA_YE$, $A_YE(AB_{VA})E$, $(A_WB_{VA})E(A_YB)E$, and $(A_YB)E(AB_{VA})E$.

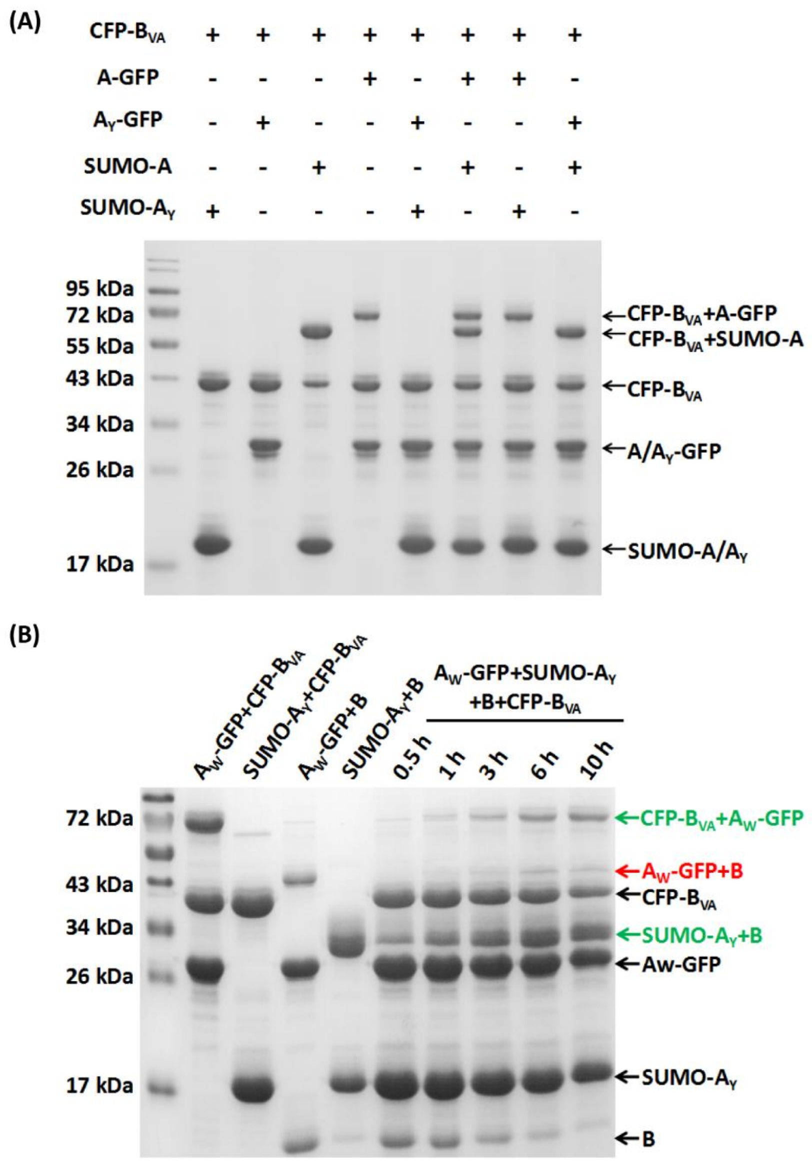

Figure S24. (A) The SDS-PAGE analysis clearly shows that no products were formed between CFP-B_{VA} and any telechelic protein containing A_Y after reaction at 4 ºC for 5 hours (the molar ratio is 1:1 at the concentration of 30 μM each). (B) The SDS-PAGE analysis of the products from the one-pot reaction in a mixture of A_W-GFP, SUMO-A_Y, CFP-B_{VA} and B at different times at 4 ºC. The model reactions between each different combination of reactants were also shown for comparison. The reaction concentration is 30 μM for CFP-B_{VA} or B and 60 μM for A_W-GFP or SUMO-A_Y. The cross-reaction products formed only at later stages of reaction in very small amounts.

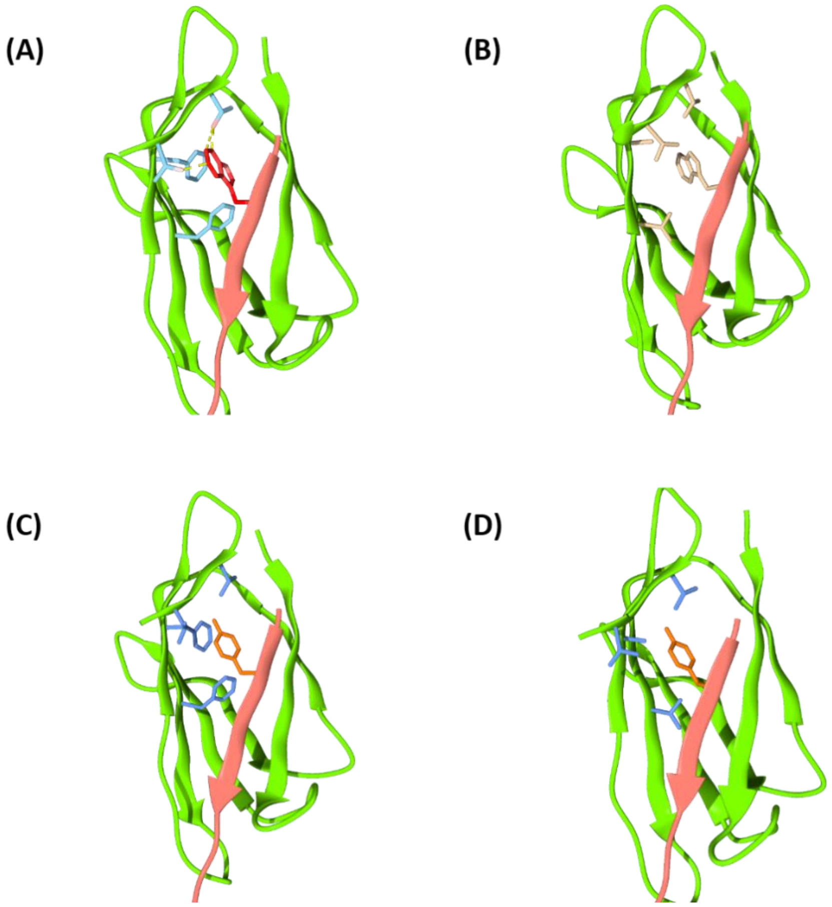

Figure S25. Structure models of the SpyTag-SpyCatcher mutant pairs: **(**A, B) Models of (A_W:B) and (A_W:B_{VA}). Trp3 in A_WB cannot form good hydrophobic interactions and clashes with other residues in B. The yellow dashed lines indicate that atomic distances are less than 3.5 Å; (C, D) Models of (A_Y:B) and (A_Y:B_{VA}). Model of (A_Y:B) has a better packing score (0.758) than (A_Y:B_{VA}) (0.683).

References

1. X. W. Wang and W. B. Zhang, *Angew. Chem. Int. Ed.* , 2016, **55**, 3442-3446.
2. T. H. Yoo and D. A. Tirrell, *Angew. Chem. Int. Ed.*, 2007, **46**, 5340-5343.
3. J. O. Fierer, G. Veggiani and M. Howarth, *Proc. Natl. Acad. Sci. USA*, 2014, **111**, E1176-1181.
4. C. Tan, P. Marguet and L. You, *Nat. Chem. Biol.*, 2009, **5**, 842-848.
5. L. Zheng, U. Baumann and J.-L. Reymond, *Nucleic Acids Res.*, 2004, **32**, e115-e115.
6. S. C. Reddington and M. Howarth, *Curr. Opin. Chem. Biol.*, 2015, **29**, 94-99.
7. P. S. Huang, Y. E. Ban, F. Richter, I. Andre, R. Vernon, W. R. Schief and D. Baker, *PLoS One*, 2011, **6**, e24109.
8. L. Li, J. O. Fierer, T. A. Rapoport and M. Howarth, *J. Mol. Biol.*, 2014, **426**, 309-317.
9. P. Conway, M. D. Tyka, F. DiMaio, D. E. Konerding and D. Baker, *Protein Sci.*, 2014, **23**, 47-55.
10. S. Lewis and P. B. Stranges, Documentation for InterfaceAnalyzer application, https://www.rosettacommons.org/manuals/archive/rosetta3.4_user_guide/df/dc8/_interface_analyzer_doc.html, (accessed July 12, 2017).
